# Transcription-factor occupancy at HOT regions quantitatively predicts RNA polymerase recruitment in five human cell lines

**DOI:** 10.1186/1471-2164-14-720

**Published:** 2013-10-20

**Authors:** Joseph W Foley, Arend Sidow

**Affiliations:** 1 Department of Genetics, Stanford University, 300 Pasteur Drive, Stanford, California 94305, USA; 2 Current address: Douglas Mental Health University Institute, McGill University, 6875 Boulevard LaSalle, Verdun, Québec H4H 1R3, Canada; 3 Department of Pathology, Stanford University, 300 Pasteur Drive, Stanford, California 94305, USA

**Keywords:** Transcription factor, ChIP-seq, HOT region, Gene regulation

## Abstract

**Background:**

High-occupancy target (HOT) regions are compact genome loci occupied by many different transcription factors (TFs). HOT regions were initially defined in invertebrate model organisms, and we here show that they are a ubiquitous feature of the human gene-regulation landscape.

**Results:**

We identified HOT regions by a comprehensive analysis of ChIP-seq data from 96 DNA-associated proteins in 5 human cell lines. Most HOT regions co-localize with RNA polymerase II binding sites, but many are not near the promoters of annotated genes. At HOT promoters, TF occupancy is strongly predictive of transcription preinitiation complex recruitment and moderately predictive of initiating Pol II recruitment, but only weakly predictive of elongating Pol II and RNA transcript abundance. TF occupancy varies quantitatively within human HOT regions; we used this variation to discover novel associations between TFs. The sequence motif associated with any given TF’s direct DNA binding is somewhat predictive of its empirical occupancy, but a great deal of occupancy occurs at sites without the TF’s motif, implying indirect recruitment by another TF whose motif is present.

**Conclusions:**

Mammalian HOT regions are regulatory hubs that integrate the signals from diverse regulatory pathways to quantitatively tune the promoter for RNA polymerase II recruitment.

## Background

Transcription factors (TFs) are proteins that regulate the expression of genes by binding the DNA at their regulatory elements (promoters or enhancers) and either preventing or facilitating the recruitment, in eukaryotes, of the transcription preinitiation complex (PIC). The PIC in turn recruits RNA polymerase II (Pol II) to the transcription start site (TSS) to synthesize an RNA transcript. This is a primary mechanism for the regulation of gene expression in response to environmental stimuli or developmental programs.

Promoters must integrate a multitude of signals that converge on them in the form of activating or repressing transcription factors. In invertebrates, some regulatory regions ("high-occupancy target", or HOT, regions) are occupied by a large number of transcription factors [1-6]. However, less is known about the interactions among TFs at HOT regions and how these interactions contribute combinatorially to the regulation of transcription, and until recently, insufficient data existed to search for HOT regions in human cells.

The ENCODE data set [7,8] provides the first opportunity to study a large group of TFs together in human cells. These data come from the chromatin-immunoprecipitation sequencing (ChIP-seq) protocol: chromatin is crosslinked to preserve DNA-protein and protein-protein bonds, then a target-specific antibody is used to capture the DNA proximally associated with a given protein, and this DNA is sequenced and aligned to a reference assembly to create a genome-wide map of protein occupancy [9]. At each genome site occupied (though not necessarily bound directly) by a protein, ChIP-seq produces a tight cluster of read alignments, which can then be detected by software with high resolution.

Previous ChIP-seq analyses have generally considered a single experiment at once, and have treated TF occupancy as a binary signal—present vs. absent. However, the particular strength of the signal at any given site may represent important biological information, such as the persistence of occupancy within a cell or frequency across all cells in the sample. We developed a new software package, UniPeak, to analyze these data accordingly.

Using UniPeak to discover and quantify HOT regions, we performed a comprehensive analysis of these regulatory hubs. In particular, we characterized HOT regions with regard to other known markers of gene activity. We also compared the occupancy profiles of different TFs to predict novel interactions, and used mechanistic evidence to infer which complex members directly bind DNA. Finally, we quantified the relationship between TF occupancy and several measures of gene expression at HOT promoters.

## Results

### The human genome contains thousands of HOT regions

We obtained all publicly available ENCODE ChIP-seq data from the 5 most studied human cell lines [8], which assayed 96 DNA-associated proteins (Additional file 1: Table S1). These cell lines are derived from a variety of tissues and germ layers: GM12878 (lymphoblastoid/mesoderm), H1-hESC (embryonic stem cell), HeLa-S3 (epithelium/ectoderm), HepG2 (hepatic/endoderm), and K562 (leukocyte/mesoderm). We aligned the read sequences from each experiment to the hg19 reference genome, standardizing the read length and removing low-confidence alignments in order to ensure accurate mapping without read-length bias.

**Figure 1:**
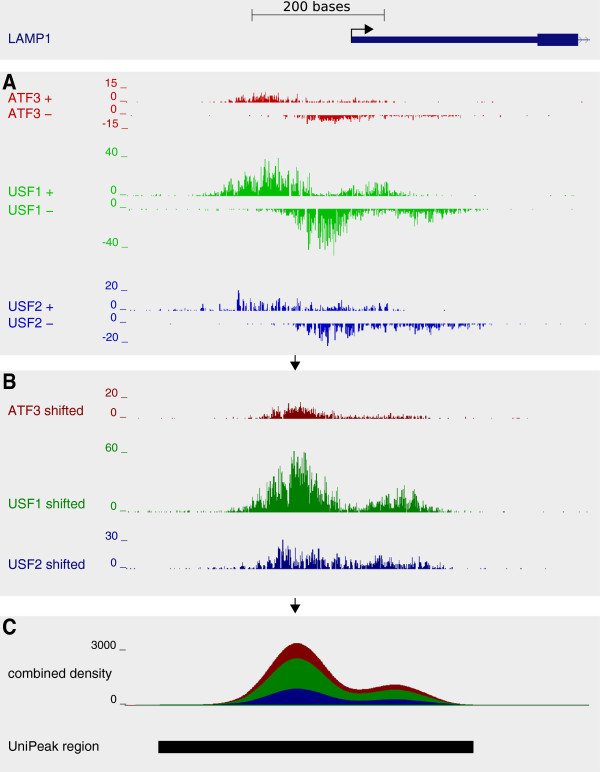
**The** **UniPeak****workflow.** **A**: Sequence reads are considered as hits at their 5^′^ start positions, strand-specifically. **B**: A global read-shift value is computed independently for each sample to align forward and reverse reads. **C**: The shifted reads from all samples are then used to estimate a single underlying density profile. Enriched regions are identified where this density exceeds a fixed threshold, determined as a function of sequencing depth and genome size. Shifted reads from each sample are counted within these regions, providing a read count for each sample within each genomic region.

UniPeak extends the peak-calling algorithm from QuEST[10] to the parallel analysis of multiple samples (Figure 1). Each aligned sequence read is considered one hit at the 5^′^ end of its alignment to the reference assembly. For each sample (i.e. a single replicate of a single experiment), UniPeak estimates the base-pair shift between strands, due to reading from opposite ends of sheared fragments, by selecting a shift value that maximizes strand correlations at the strongest regions. After shift correction of individual samples, kernel density estimation is used to compute a single smooth density profile for the combined signal of all samples. UniPeak identifies enriched regions where this profile exceeds a fixed threshold of fold enrichment relative to a uniform background distribution. The number of hits within each of these regions from each sample is reported, yielding a regions × samples matrix of hit counts. Unlike other peak-callers for ChIP-seq, UniPeak does not directly use "input" or other negative controls to filter enriched regions initially; rather, though these samples do not contribute to the region-calling step, negative-control reads (as well as histone-mark ChIP reads) are counted within the regions called from ChIP samples, and reported alongside read counts from TF ChIP samples. We normalized the peak intensities from discrete read counts to continuous occupancy values with the variance-stabilizing transformation in DESeq[11]. Performance validation of UniPeak is described in Additional File 2.

**Figure 2:**
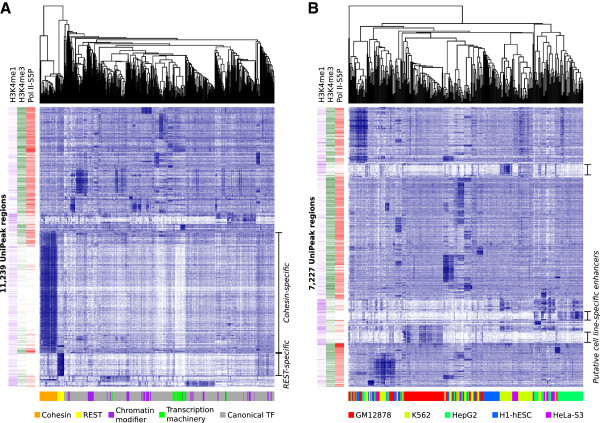
**Clustering of protein occupancy data.** **A**: Hierarchically clustered heatmap of all DNA-associated proteins assayed. Each row represents a HOT region called on the pooled data by UniPeak (*m* = 11,239), and each column represents a single replicate of a ChIP-seq experiment (*n* = 503). The sidebars show histone modification ChIP-seq signals within the same regions, normalized separately, and initiating RNA polymerase II signal and the nearest peak within 500 bp (zero if there were no peaks in this range). Color intensity in the heatmaps corresponds to variance-stabilized values for occupancy. **B**: Canonical TFs only (7,227 regions × 357 samples).

From the full set of 96 proteins in 5 cells, UniPeak detected 11,239 enriched regions (Table 1) of median size 136 bp (Additional file 1: Figure S3). Many of these appeared roughly evenly occupied by most proteins, with notable exceptions (Figure 2A). In particular, a large fraction of these regions were occupied only by the cohesin complex (CTCF, RAD21, SMC3), which, unlike canonical TFs, is known to bind insulator elements [12]. Cohesin-specific sites were less likely to be near a Pol II initiation site, and showed depletion of histone 3 lysine 4 trimethylation (H3K4me3), a chromatin mark associated with active promoters [13]. REST, a transcription repressor that binds the RE1 element to repress neuronal genes in non-neurons [9,14-16], similarly showed preferential occupancy in a large set of regions depleted for other TFs and for initiating Pol II.

**Table 1:** Results of region calling

**Data set**	**Proteins**	**Samples**	**Reads**	**UniPeak****regions**	**Reads in regions**	**RNA polymerase II initiation sites**	**CAGE peaks**	**RefSeq TSS**	**Consensus promoters**
All proteins	96	503	8.8B	11,239	213M (2%)	5,631 (50%)	4,951 (44%)	4,703 (42%)	4,189 (37%)
TFs only	75	357	6.3B	7,227	118M (2%)	5,745 (79%)	5,128 (71%)	4,813 (67%)	4,441 (61%)
GM12878	46	102	1.8B	12,887	61M (3%)	7,477 (58%)	6,315 (49%)	5,011 (39%)	4,522 (35%)
K562	41	93	1.5B	14,578	70M (5%)	10,174 (70%)	7,188 (49%)	6,589 (45%)	5,743 (39%)
HepG2	32	76	1.5B	12,312	48M (3%)	6,557 (53%)	5,232 (42%)	4,199 (34%)	3,791 (31%)
H1-hESC	25	52	985M	3,392	8M (1%)	2,180 (64%)	2,303 (68%)	2,127 (63%)	1,700 (50%)
HeLa-S3	16	34	498M	13,199	18M (4%)	5,499 (42%)	4,056 (31%)	3,243 (25%)	2,893 (22%)

To focus on only canonical TFs, which should be more functionally homogeneous and increase our specificity for HOT regions, we removed from the analysis four classes of proteins with different behaviors that confounded our goal of HOT region analysis. These were the cohesin complex, REST, chromatin remodelers and modifiers (e.g., p300 and SWI/SNF), and the preinitiation complex. The latter was later used to test functional predictions.

With this reduced set of 75 canonical TFs, UniPeak detected 7,227 regions (Table 1), of median size 171 bp (Additional file 1: Figure S3). Consistent with HOT regions, these regions were occupied by most or all TFs (Figure 2). Hierarchical clustering showed that the occupancy profiles of different TFs in the same cell were generally more similar than those of the same TF across all cells. In particular, GM12878, K562, and HepG2 each showed sets of HOT regions that were only occupied in one cell type, and these tended to be depleted for initiating Pol II and for histone 3 lysine 4 trimethylation vs. monomethylation; these regions might represent cell line-specific enhancers.

Because of these cell-specific signals and because of the incomplete overlap among the sets of TFs tested in different cells (Additional file 1: Table S1), we also used UniPeak to detect enriched regions in each of the 5 cell lines individually. This yielded 12,312–14,578 HOT regions from each data set, except H1-hESC with only 3,392 (Additional file 1: Figure S4). The generally higher number of detected regions may reflect higher sensitivity to cell-specific binding than in the pooled analysis, and a general lack of active cell-specific sites in H1-hESC (perhaps differentiated lineage-specific enhancers, since H1-hESC showed much higher promoter enrichment (50% consensus promoters vs. 22–39% in other cell types); this is consistent with a model in which tissue-specific enhancers are inactive or "poised" in undifferentiated cells [17]).

### Many HOT regions are promoters

Since transcription factors occupy regulatory elements in the genome, we expected HOT regions to align with these elements. We compared the positions of these HOT regions with those of known or inferred promoters, according to three lines of evidence. First, we detected initiating RNA polymerase II (serine 5-phosphorylated [18]; Pol II-S5P) enrichment sites from an independent UniPeak analysis, again using ENCODE ChIP-seq data. Second, we used a strand-specific UniPeak analysis to detect enriched regions from CAGE, a form of RNA-seq that captures short tags at the 5^′^ end of the transcript [19]. Finally, we used transcription start site (TSS) positions from RefSeq [20], the most robust and most stringent annotation.

**Figure 3:**
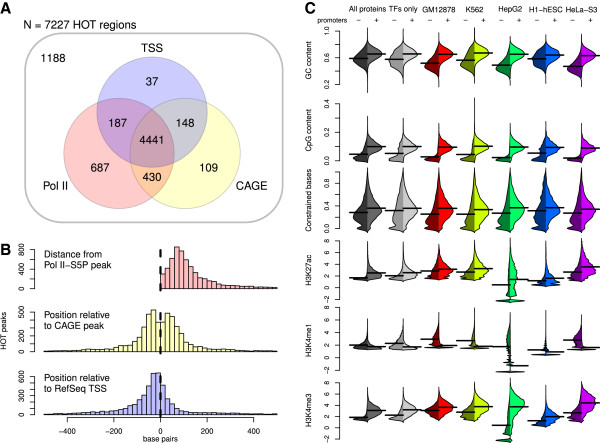
**Characterization of HOT regions with reference annotations.** **A**: Overlap among promoter annotations. Each HOT region from the analysis of all canonical TFs in all cells (*N* = 7,227) was annotated with the nearest initiating RNA polymerase II peak, CAGE peak, or RefSeq TSS within 500 bp. **B**: Proximity of TFs’ binding regions to promoters, in base pairs, according to nondirectional RNA polymerase II ChIP-seq data, directional CAGE data, or RefSeq annotations of transcription start sites. **C**: Quantitative annotations versus presence of Pol II. Each row of figures shows distributions of a given annotation in the two combined and five cell-specific data sets, separated between HOT regions not at a consensus promoter (dark) vs. regions within 500 bp of a Pol II binding site, a CAGE peak, and a RefSeq TSS (light).

Of the 7,227 HOT regions called using the set of canonical TFs in all cells, 79% were within 500 bp of Pol II-S5P occupancy peaks, 71% within 500 bp of CAGE enrichment peaks, and 67% within 500 bp of RefSeq TSSs, with 61% "consensus promoters", i.e. within 500 bp of all three features (Figure 3A). Of HOT regions with occupancy peaks within 500 bp of one of these annotations, most fell within 200 bp of the given annotation (83% Pol II-S5P, 88% CAGE, 85% RefSeq TSS), with a bias toward being upstream rather than downstream of annotated TSSs (68% upstream; Figure 3B). Among the regions called in the five cell-specific analyses, 42–70% were near Pol II-S5P sites, 31–68% near CAGE peaks, and 25–63% near annotated TSSs (Table 1); the variation in these ranges reflects the different sets of TFs tested in the different cells. RefSeq TSS was consistently the least common annotation, perhaps because the database represents an incomplete set of true promoters, whereas Pol II-S5P ChIP-seq and CAGE enrichment signals occur at active TSSs regardless of whether they are annotated.

We further characterized HOT regions at consensus promoters in terms of several quantitative genomic features associated with promoters (Additional file 1: Figure S5). Most known human promoters are enriched for GC content and especially CpG dinucleotides [21], and their sequences are typically under evolutionary constraint [22]. In addition, several histone modifications are associated with regulatory genome elements: histone 3 lysine 4 trimethylation (H3K4me3) is enriched at active promoters [13], while monomethylation (H3K4me1) is enriched at enhancers [23], and histone 3 lysine 27 acetylation (H3K27ac) is enriched at both active promoters [24,25] and active enhancers [17,25,26]. Consistent with active promoters, our consensus promoters showed higher GC content, CpG content, evolutionary constraint, H3K27ac, and H3K4me3 vs. H3K4me1 (Figure 3C). Since these regions showed strong evidence of being promoters and could be associated with specific genes, we restricted all subsequent analyses to the consensus promoters in each of the five cell-specific HOT region lists, treating them as independent replicate experiments.

### Similar occupancy profiles suggest binding partners

**Figure 4:**
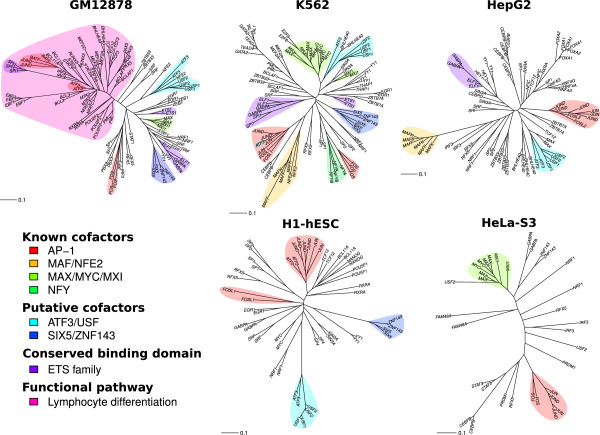
**Neighbor-joining trees for TFs at consensus promoters in the cell-specific analyses.** Clustering is by Pearson distance of occupancy profiles. Individual replicates are shown separately. Known and putative functional groups are highlighted.

We reasoned that TFs with correlated occupancy profiles (more abundant at some sites and less abundant at others) may share mechanistic or functional relationships. To search for such relationships, we used neighbor-joining [27] to cluster TFs by the similarity of their occupancy profiles across consensus promoters in each cell (Figure 4). This analysis detected some known binding partners and gene families as well as novel relationships among TFs. For example, subunits of multimeric complexes often had very similar binding profiles, such as NFE2 and MAFF or MAFK [28]; MAX and MYC or MXI1 [29,30], NFYA and NFYB [31,32]; and USF1 and USF2 [33]; to a lesser extent, so did family members that share a DNA-binding motif, such as the ETS family (ELF1, ETS1, GABPA, SPI1) and the E-box family (MYC, USF1, USF2).

The AP-1 transcription factor is a heterodimer composed of a member of the JUN family and a member of either the FOS family or the ATF family [34]. However, in our analysis, FOS itself never clustered with a JUN family member, but JUN and JUND’s occupancy profiles were correlated with those of their alternative binding partners BATF, FOSL1, FOSL2, and ATF2, and to a lesser extent CEBPB and CEBPD, which are not documented to interact with AP-1 subunits. Unlike ATF2, ATF3 reproducibly clustered with USF1 and USF2, which have no documented interaction with ATF3. The occupancy profiles of SIX5 and ZNF143 were also correlated in multiple cell types despite no documented interaction. In fact, mammalian two-hybrid assays found no direct binding activity between these proteins [35].

One large branch in GM12878 included ATF2 [36], BATF [37], BCL3 [38], BCL11A [39], BCLAF1 [40], BHLHE40 [41], EBF1 [42], IRF4 [43], JUND [44], MEF2C [45], MTA3 [46], NFATC1 [47], PAX5 [48], PML [49], POU2F2 [50], RUNX3 [51], RXRA [52], SPI1 [53], STAT3 [54], STAT5A [55], TCF3 [56], and TCF12 [57], which are all known to be involved in the differentiation of lymphocyte lineages. This branch also included MEF2A, which, unlike its highly similar family member MEF2C, is not known to be involved in lymphocyte differentiation [58]. Thus, this analysis both recovered known functional relationships between TFs and discovered novel associations.

To test whether protein-protein interactions predict similarites in occupancy patterns, we compared our results with a comprehensive database of mammalian two-hybrid screens; data were available for all TFs in this study except FAM48A and THAP1 [35] Within each cell type, we split pairwise correlations of samples’ occupancy profiles across all HOT regions into those from binding TF pairs and those from non-binding TF pairs. Pairs of replicates of the same TF were not used. On average, the occupancy profiles of binding TFs were more correlated than those of non-binding TFs (Additional file 1: Figure S6). The difference was only large in the HeLa-S3 data, perhaps due to the selection of TFs tested in that cell type; in other words, potential direct interactions between TF pairs (which may not actually occur *in vivo*) generally only explain a small part of the similarity in their occupancy patterns.

### Most TFs appear to be recruited to HOT regions as cofactors

Although it is difficult to use shared occupancy profiles to infer a binding mechanism, additional analysis can illuminate a critical step in the recruitment of a TF complex. A TF’s observed occupancy at a given promoter might be due to either direct binding of DNA or recruitment by another protein. Most TFs in our data set have previously been annotated with DNA sequence motifs that they bind specifically. Thus, if we make the simplifying assumption that most TFs usually bind DNA at regions that contain their respective sequence motifs, then their occupancy at sites without their motifs is likely to be as cofactors recruited by other proteins.

**Figure 5:**
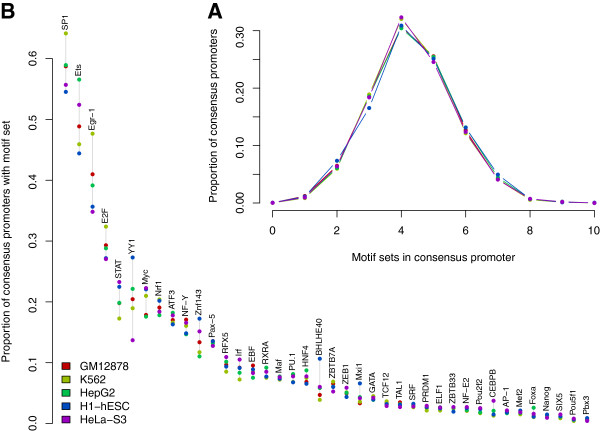
**Frequency of DNA sequence-motif sets.** Each set contains one or more motifs annotated to the same TF. **A**: Number of distinct motif sets represented at each consensus promoter peak. **B**. For each motif set, the number of consensus promoter peaks with at least one motif from the set.

To identify candidates for direct DNA binding, we searched across the consensus promoters from the cell-specific UniPeak output for occurrences of sequence motifs associated with the TFs in the ENCODE data set. We considered motifs identified *de novo* by ENCODE from analysis of each ChIP-seq experiment individually (Kheradpour P, Kellis M: ENCODE-motifs: systematic analysis of regulatory motifs associated with transcription factor binding in the human genome, under revision), and in order to avoid motifs that are not bound directly by a given TF but rather by its cofactor, we used only motifs that matched database annotations for the given TF. This yielded multiple motifs for some TFs and none for others, and some motifs were associated with TF families rather than individual TFs; thus, our analysis was based on "motif sets" that share a common annotation, rather than individual motifs. On average, any given HOT promoter contained motifs in about 4 distinct sets (Figure 5A), even though these sites are defined by the presence of many more TFs, suggesting that the majority of TFs at these sites may be recruited by other factors rather than bound to the promoter themselves. Furthermore, the number of occurrences of any set’s motifs across the set of regions was too small for nearly any particular TF to bind its motif at most HOT regions (Figure 5B).

**Figure 6:**
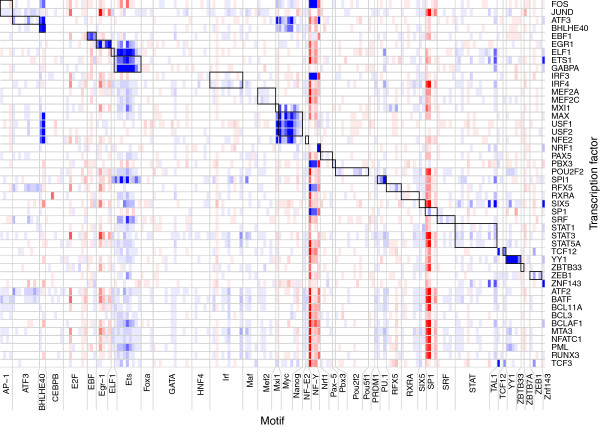
**Predictive value of all motifs for all TFs, in GM12878 cells.** Each row represents a single TF, with replicates pooled. Each column represents one sequence motif from the previous analysis, and motif sets associated with the same TF are grouped. Color intensity represents the significance of the difference in occupancy of the given TF at consensus promoters with vs. without the given motif, where significance is the logarithm of the *p*-value from a *t*-test for the difference between means of occupancy scores, which are the means of variance-stabilized scores across replicates. Blue signifies enrichment of the given TF at sites with the given motif, and red signifies depletion of the given TF at sites with the given motif.

Next we measured the association between motifs and TF occupancy at consensus promoters. For each motif, we compared the occupancy score of each TF between consensus promoters with the motif vs. those without it Figure 6, (Additional file 1: Figure S7). Many motif sets were predictive of the occupancy of other TFs besides their own, and some TFs’ occupancy was better predicted by other TFs’ motifs than by their own. The most promiment pattern was that ETS-family motifs were strongly predictive of many other TFs’ occupancy. In particular, consensus promoters with ETS motifs were enriched for the occupancy of MAFF, MAFK, MEF2A, MEF2C, POU2F2, and SRF, suggesting that these TFs’ primary mechanism of positioning at HOT regions may be recruitment by ETS family members rather than direct DNA binding.

Motifs in the E-box family are bound by TFs with a basic helix-loop-helix domain, including BHLHE40 [59], MYC-MAX [29], MXI1-MAX [30], TCF12 [60], and USF1/USF2 [33]. Other TFs enriched at E-box sites included ATF3, E2F6, NFE2, and SIN3A; of these, the only previously documented interaction with a E-box-binding TF is between SIN3A and MAD-MAX [61].

Subunits of the AP-1 transcription factor were only weakly enriched at promoters containing motifs for the FOS-JUN heterodimer; however, JUN and JUND, but not FOS, were more strongly enriched at sites with motifs associated with their alternate binding partner ATF3. The ATF3 motifs were also predictive of the occupancy of CEBPB, RFX5, and SRF, none of which are documented to interact with AP-1 directly; however, although neither is enriched at the other’s motif sites, CEBPB and SRF are known binding partners [62,63]. On the other hand, FOS, but not FOSL1 or FOSL2, was very strongly enriched at sites with the NF-Y motifs, as were IRF3 (but not IRF4), PBX3, RFX5, SP1, and SP2. Of these, only SP1 is known to interact with NFYA/NFYB [64].

Other relationships between a motif set and TFs not annotated with it include one particular MAF motif with SPI1; the NRF1 motifs with ATF3; certain STAT motifs with ELF1, ETS1, SIX5, SPI1, and ZNF143; TAL1 motif with TCF3 and TCF12; and ZNF143 motif with ETS1 and SIX5. Of these relationships, all but the last can be explained by motif sequence similarity; no interaction among ZNF143, ETS1, and SIX5 is documented. Some of the most common motifs, the GC-rich EGR1 and SP1 sets, were associated with depletion of most TFs. The NRF1 and NF-Y motifs were associated with depletion of many TFs except the few that were strongly enriched at those sites.

The occurrence of TF-associated DNA sequence motifs in HOT regions was so low, relative to the number of TFs present, that most TFs probably do not directly bind the DNA at these regions but are instead recruited as cofactors, consistent with other analyses of these data [65]. Reinforcing this, many TFs’ occupancy was well predicted by motifs known to be bound directly by different TFs, and in some cases a TF showed a stronger preference for a different TF’s motif than for its own.

These results are corroborated by a previous analysis of the same data [66]; however, most of the putative transcription-factor interactions inferred in that analysis are not supported by ours. Our analysis may be more stringent because it considers the strength of the ChIP-seq signal at each site rather than just presence or absence of a peak called at arbitrary thresholds.

### A small number of TFs explain a large proportion of Pol II recruitment

The general role of TFs is to recruit the preinitiation complex and ultimately Pol II, which then transcribes RNA from the gene body; thus, the presence of these downstream factors and the abundance of the transcript should be partially explained by the combination of TFs at promoters. We also expect a relationship between TF occupancy and histone modifications associated with active promoters, though the causality may work in either direction. Since we have quantitative enrichment values for all these markers of gene regulation and for all TFs’ occupancy, at all consensus promoters, we can measure the strength of the relationship between them statistically.

We constructed a linear regression model that treated each TF ChIP-seq sample as an independent variable, and gene regulation as the dependent variable, with each HOT consensus promoter as one observation. This model necessarily contains redundant signals, not just as strong correlations between replicates, but also as weaker correlations between factors with similar behaviors, such as sets of TFs that bind in complex; because of the number of predictors and their nested multicollinearity, standard multiple linear regression would produce uninterpretable results and suffer from overfitting or reduced power. We instead applied partial least-squares regression, which performs a rotation and dimensional reduction on the covariance matrix in order to isolate latent orthogonal signals underlying patterns from multiple observations. This method also allows both the independent and dependent variables to be matrices rather than single vectors, so only two models (v.i.) were fit for each cell type, encompassing all available data at once. The dependent variables we used were the occupancy of PIC subunits within the region, the enrichment of histone modifications (H3K4me1, H3K4me3, H3K27ac) within the region, the occupancy of initiating Pol II at its nearest enriched region, the occupancy of elongating Pol II (serine 2-phosphorylated [18]; Pol II-S2P) in the gene body corresponding to the nearest RefSeq TSS, the CAGE signal at its nearest enriched region, and the RNA-seq signal for the gene corresponding to the nearest RefSeq TSS. Since all the signals from the available experiments were required for a full observation, this analysis was restricted to consensus promoters; genes with no TFs bound were not used to train the model.

As a null model, we considered that any explanatory power from the TF signals that could also be contributed from "input" controls (total chromatin, IgG pulldown) was likely a ChIP-seq artifact rather than a meaningful TF effect. Therefore, for each cell type we compared two models: gene regulation as a function of both TF ChIP and input signal, and gene regulation as a function of input signal alone.

**Figure 7:**
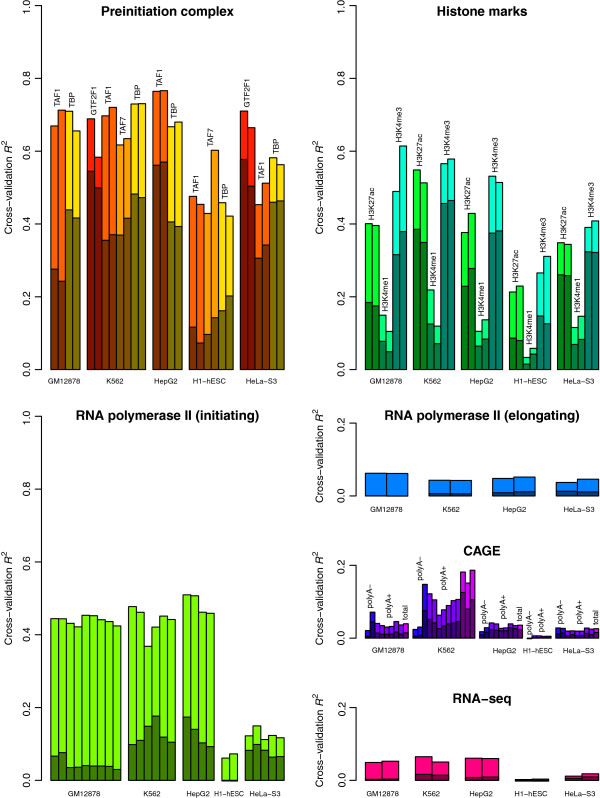
**Functional analysis of TF binding.** For each cell type, a model was fit to predict various measures of gene regulation as a function of the occupancy of all tested TFs. Cross-validation *R*^2^ values are shown separately for each replicate. Light-colored bars correspond to the full ∑ TFs + input models, dark-colored bars to the input-only models.

The presence of PIC subunits was well predicted by aggregated TF occupancy (Figure 7, Additional file 1: Figure S10; cross-validation *R*^2^ ≈ 0.7 for the cells with the most TFs tested), though with somewhat high contribution from input alone. Histone marks H3K4me3 and H3K27ac were somewhat well predicted (CV *R*^2^ ≈ 0.4), but with even higher relative contribution from input, perhaps because these controls are sensitive to open chromatin, which is associated with active promoters; H3K4me1 was not well predicted by the model (CV *R*^2^ < 0.2), likely because of very low signal at these regions, as expected for a mark depleted at active promoters. Pol II-S5P occupancy was also well predicted by TF occupancy (CV *R*^2^ ≈ 0.4), and input was not very predictive (CV *R*^2^ < 0.2); the results were slightly worse in the cells with fewer TFs tested. On the other hand, Pol II-S2P occupancy was not well predicted by TF occupancy, nor was transcript abundance as measured by either CAGE or RNA-seq (CV *R*^2^ < 0.2); there was no consistent difference between CAGE signals from polyadenylated (mature) and unpolyadenylated transcripts. Thus we found that the presence of these TFs is strongly associated with immediately subsequent steps in gene regulation, but only weakly associated with later steps.

## Conclusions

We present a quantitative analysis of a large volume of ChIP-seq data, constituting the genome-wide occupancy profiles of a large number of TFs in five human cell types, from the ENCODE consortium [8]. The new software package UniPeak facilitates the comparison of binding profiles from an unlimited number of samples at a consistent set of genome regions, eliminating the difficulty of reconciling many independent lists of peak calls and producing a regions × samples matrix of signal strengths, similar to those generated by microarray experiments. Here we bring matrix analysis and sample clustering back to the forefront of a high-throughput genomics investigation. Since we view DNA-associated protein occupancy as a fundamentally quantitative phenomenon, which may have quantitative functional effects [2], we avoid applying premature thresholds and dequantification of the peak intensities. Our approach may become even more useful as improved technology allows greater sequencing depths and therefore higher quantitative precision, and perhaps also as new molecular protocols increase the signal-to-noise ratio of protein-associated DNA capture [67].

Assessing the relevance of this study to our understanding of transcriptional regulation, we found that about 40% of variance in initiating Pol II occupancy at HOT promoters can be explained by the entire set of available TF occupancy data in the cells with the most experiments. The predictive value is higher for PIC subunits, and much lower for elongating Pol II and transcript abundance. These results are also consistent with our knowledge of biological mechanisms, because there are many additional regulatory interactions between PIC recruitment and the production of an elongated, mature, stable transcript that do not involve TFs. It is important to note that these models would have shown a better fit if we had surveyed all promoters instead of just those occupied by many TFs, because the inclusion of inactive promoters would add many points near the origin (no TFs bound, no gene expression), which would make the trend more linear [7,68-70]. Finally, this analysis represents fewer than 50 TFs tested in any individual cell line, compared with the 1,400–1,900 TFs estimated to exist in the human genome [71]; in that context, 40% of variance explained represents substantial explanatory power.

**Figure 8:**
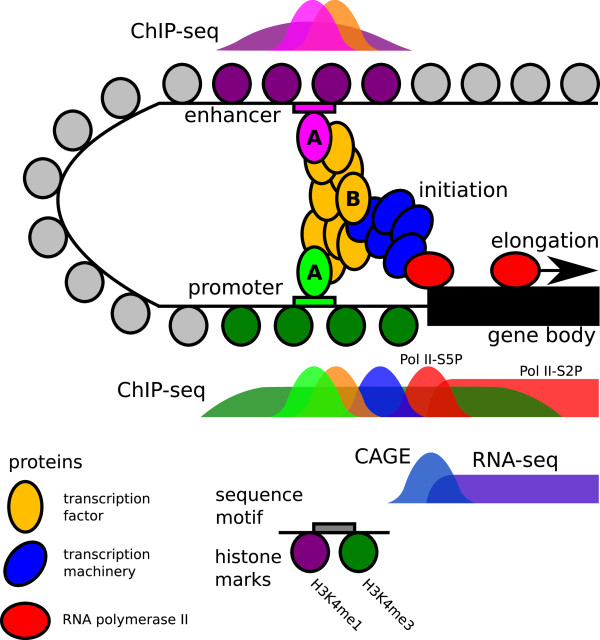
**Proposed model of interaction between two HOT regions and resulting signals from high-throughput sequencing experiments.** An enhancer is looped over and connected by a complex of many TFs, which recruit the preinitiation complex and ultimately RNA polymerase II to the TSS. Proteins A are directly bound to DNA, while protein B is recruited to the site through only protein-protein interactions. These mechanisms are indistinguishable from the ChIP-seq signal, but presence of a known sequence motif suggests which protein binds directly.

Regions occupied by many different TFs are common in the human genome. Even our strictest definition finds several thousand HOT promoters, likely a considerable fraction of the active genes in any given cell line. Especially since there are far too few known DNA sequence motifs to account for all the TF occupancy at these sites, we propose that TFs collaborate combinatorially through protein-protein interactions to regulate Pol II recruitment (Figure 8), concordant with similar evidence from *Drosophila* enhancers [72]. Interactions of this nature have not previously been examined on such a large scale, due to the greater challenges of high-throughput peptide assays compared to high-throughput nucleotide assays.

This analysis yields several hypotheses that may be validated by future experiments. Based on the similarity of their occupancy profiles, reproduced independently in multiple cell lines, we infer that ATF3 and USF1/USF2 may be part of a novel protein complex; furthermore, since DNA sequence motifs associated with USF1/2 are predictive of ATF3’s occupancy but not vice versa, we predict that USF1 or USF2 is the subunit of this complex that directly binds the promoter, while ATF3 is a cofactor recruited by USF1/2 despite having its own DNA-binding domain that is used in other complexes. By the same logic we also predict that SIX5 and ZNF143 are members of a novel complex in which ZNF143 is the DNA-binding subunit. In both cases, previous experimental evidence shows the two partners are not capable of binding each other alone, suggesting that a chaperone is required to enable binding, or that these interactions require both proteins to bind to a common intermediate or complex of intermediates. On the other hand, we find that in HOT promoters, FOS seems only rarely to perform its well-known role in the AP-1 heterodimer with JUN, generally supplanted by a FOSL or ATF protein, though of course FOS is known to have several alternate cofactors [34].

It should not be surprising that a TF with a functional DNA-binding domain and even a well-demonstrated sequence motif might often be recruited as a cofactor by some other protein. Indeed, this is true of TBP, the so-called TATA-binding protein, which is required for the assembly of the preinitiation complex at all loci even though only 10–24% of human promoters have a TATA box [73,74]. One possible paradigm for gene-regulatory evolution might be the emergence of a DNA-binding TF that uses protein-protein interactions to recruit other TFs to its own recognition sites, harnessing their existing regulatory pathways without sequence motifs for the other TFs. Over evolutionary time, this additional layer of regulatory interactions between the steps of protein-DNA binding and recruitment of polymerase might remove the constraint of requiring the "downstream" TFs’ sequence motifs in new regulatory elements or even conserving them in existing elements, so that TFs capable of autonomously binding DNA and recruiting the PIC become primarily cofactors for other TFs with more specialized target loci and finer regulatory control. Thus the large TF complexes, or interchangeable interactions, that we observe at HOT regions might represent multiple levels of gene regulation and therefore of evolutionary history.

## Materials and methods

### Read alignment

ChIP-seq nucleotide sequence reads and base qualities were obtained from the ENCODE database, and truncated to the first 25 nt, the shortest length in the data set, to prevent biases in mapping due to different read lengths. BWA 0.6.1-r104[75] was used to map reads to the hg19 reference assembly. Unique best hits were filtered to confident alignments with posterior probability ≥ 0.9.

### Density profiles

Similarly to the robust QuEST algorithm [10], a smooth density profile was created using the frequency of 5^′^ read starts per reference base as the input to kernel density estimation (KDE), so that the density at any given position *i* on one strand was given by

$$H(i)=\frac{\sum_{j=i-h}^{i+h}K\left(\frac{i-j}{h}\right)C(j)}{\sum_{k=-h}^{h}K(\frac{k}{h})}$$

where $K(x)=\frac{3}{4}(1-x^{2}){\text{1}}_{\left\{|x|\leq 1\right\}}$ is the Epanechnikov (quadratic) kernel density function, *h* is the kernel bandwidth, and *C*(*j*) gives the number of 5^′^ read starts at position *j*.

### Enriched region calling

Any region where the smooth density profile exceeded a fixed threshold, relative to the uniform background of the total confident read count divided by the genome size, was considered enriched. 5^′^ read starts were then counted inside each region. The kurtosis of the distribution of 5^′^ read starts within each region was calculated, and leptokurtic regions were filtered out to remove technical artifacts.

### Strand shift estimation

To estimate the shift between enrichment maxima from the forward and reverse strands flanking each binding site, a byproduct of 5^′^ end-directed sequencing and the genomic fragment size, KDE was performed separately on each strand, and preliminary enriched regions were called from the sum of the two density profiles. Among the regions containing the highest read counts, the Pearson correlation between the strand-specific density profiles was calculated for each of a spectrum of 5^′^ to 3^′^ shift values. The distribution of correlation-maximizing shift values across the top regions was smoothened with a small bandwidth and the global maximum was chosen as the sample-wide shift value. Density profiles from opposite strands were shifted by this value and added together for a unified, strand-independent profile. Regions with a low Pearson correlation between the two strands’ density profiles were discarded as artifacts.

### UniPeak workflow

The new software package UniPeak was written to automate the steps above. Starting with confidently aligned reads, strand shift was estimated independently for each sample (with the exception of negative controls, whose shift was inferred from corresponding ChIP samples, as they did not yield enough preliminary enriched regions to estimate a shift value), using the top 1,000 regions called with smoothing bandwidth 50 nt, region-calling fold-enrichment threshold 25X, kurtosis threshold 50, minimum strand correlation 0.3, minimum shift 25 nt each strand, maximum shift 150 nt each strand, and correlation vs. shift smoothing bandwidth 5 nt. The samples were then shifted accordingly and kernel smoothing was performed with bandwidth 100 nt to capture binding sites in close proximity to each other; density profiles from both strands of all samples were summed and enriched regions were called and filtered as before. Enriched regions on sex chromosomes and the mitochondrial genome were removed, along with those overlapping false-positive genome regions identified by ENCODE and those greater than 500 bp in size.

### Normalization

The read-count matrix from UniPeak 1.0 was normalized by the variance-stabilizing transformation in DESeq 1.7.7[11], determining the dispersion-mean relationship with local fitting, pooling all samples to estimate a single empirical dispersion value per analysis, and using only the fitted dispersion-mean relationship values. Replicate experiments from different laboratories were treated as separate classes.

### Clustering analysis

Clustering was performed on normalized data as described above. Distances were calculated with the Pearson metric (1-*r*). Rooted, ultrametric trees were generated by hierarchical clustering with UPGMA as implemented in the fastcluster 1.0.4 package in R 2.12.1[76]. Unrooted trees were generated by neighbor-joining [27] as implemented in RapidNJ 2.1.0[77].

### Comparison with annotations and independent data

Initiating RNA polymerase II ChIP-seq data were treated in the same manner as TF data, but independently from that analysis, with 50 nt smoothing bandwidth for region calling and no region size filter. A TF-enriched region was matched to a Pol II-S5P-enriched region if the maxima of the regions’ respective density profiles were within 500 bp of each other; when more than one Pol II-S5P site was near a TF site, the nearest Pol II-S5P site was used.

Transcription start site coordinates for the hg19 reference assembly were obtained from the RefSeq database [20]. A TF-enriched region was matched to a RefSeq TSS if the TSS was within 500 bp of the local maximum of the density profile within the region; when more than one TSS fell within this range, the nearest was used. Single-end, 75 nt RNA-seq reads from the ENCODE database were aligned to the hg19 RefSeq transcriptome by DNAnexus, which computed the count per transcript [78]. Elongating RNA polymerase II ChIP-seq reads were aligned to the hg19 genome, and for each annotated TSS, reads were counted between 100 bp upstream of the TSS and 100 bp downstream of the TES for the longest isoform.

CAGE reads were obtained from the ENCODE database after alignment to hg19 with Delve (T Lassmann, in prep.). CAGE-enriched regions were called via UniPeak in the same manner as TF binding sites, using 50 nt smoothing bandwidth, separate strands, and no shifting. CAGE regions were matched with TF regions in the same manner as Pol II-S5P regions.

Evolutionary constraint within a region was calculated as the proportion of positions with a rejected substitution (RS) score greater than 2, according to GERP++[22].

From ENCODE’s database we retrieved 226 sequence motifs that were both inferred *de novo* from ChIP-seq data and matched to similar motifs in other databases, such that there was a variably sized set of motifs annotated to each individual TF. These were aligned to reference sequence in a 201 bp window centered at each HOT region peak by MAST 4.6.0[79]. A HOT region peak was considered to have a hit for a given TF’s motif set if any motif in the set had a MAST hit of *E* < 10.

### Modeling gene regulation

For each cell line, we constructed a model of the general form

() $$\begin{array}{lll} \;\sum Y_{\text{PIC}} & +\sum Y_{\text{histone}}+\sum Y_{\text{pol2}}+\sum Y_{\text{CAGE}}\; & \;\\ & +\sum Y_{\text{RNA-seq}}∼\sum X_{\text{input}}+\sum X_{\text{TF}}\; & \; \end{array}$$

where the *Y* terms form a matrix of the individual replicates of the dependent variables, normalized together by DESeq as before: preinitiation-complex occupancy within the region, histone-mark occupancy within the region, Pol II occupancy at the nearest UniPeak site, CAGE signal at the nearest UniPeak site, and RNA-seq signal for the gene corresponding to the nearest RefSeq TSS; and the *X* terms form the matrix of all the individual replicates of TF occupancy scores plus the signal from negative-control samples (input, IgG, reverse-crosslinked chromatin) within the regions, normalized together. Since both the independent and depent variables were highly multicollinear, we used the pls 2.3-0 package in R 2.12.1[80] to reduce this model to latent variables by partial least-squares regression. The number of LVs used in each model was determined as the first LV plus all subsequent LVs that subtracted at least 0.01 from the average RMSEP (Additional file 1: Figures S8, S9). Cross-validation used the leave-one-out method: the *R*^2^ values were calculated by validating with each UniPeak region after re-training the model on the remainder of the data.

## Competing interests

The authors declare no conflict of interest.

## Authors’ contributions

JWF and AS conceived the study and prepared the manuscript. JWF performed all analysis. Both authors read and approved the final manuscript.

## Supplementary Material

Additional file 1 **Contains a supplementary table and supplementary figures.** All data used in this work are available from the ENCODE data portal, http://genome.ucsc.edu/ENCODE/. Scripts and processed data can be obtained at http://mendel.stanford.edu/sidowlab/downloads/hot/. Click here for file

Additional file 2 **Describes a study validating the** **UniPeak****method.** Click here for file
