# Supplementary material for: Transcription-factor occupancy at HOT regions quantitatively predicts RNA polymerase recruitment in five human cell lines
Source: BMC Genomics. 2013 Oct 20;14:720. doi: 10.1186/1471-2164-14-720 (PMC3826616; doi:10.1186/1471-2164-14-720)
Supplement: Additional file 1 — Contains a supplementary table and supplementary figures. All data used in this work are available from the ENCODE data portal, http://genome.ucsc.edu/ENCODE/. Scripts and processed data can be obtained at http://mendel.stanford.edu/sidowlab/downloads/hot/. [file 1471-2164-14-720-S1.pdf]

Table S1: ChIP-seq samples used in this analysis. Most TFs were not tested in most of the 5 cell lines. Symbols indicate which laboratory performed the experiment: M, Myers (HudsonAlpha); S, Snyder (Yale/Stanford); B, Bernstein (Broad); the number of symbols shows the number of replicates. Different antibodies to the same protein (e.g. MafK_ab50322, MafK_SC-477) showed very similar results and therefore were not treated distinctly for analysis.

	GM12878	H1-hESC	HeLa-S3	HepG2	K562
	canonical transcription factor				
ATF2	MM	MM			
ATF3	MM	MM		MM	MM
BATF	MM				
BCL11A	MM	MM			
BCL3	MM				
BCLAF1	MM				MM
BHLHE40	SS			MMSS	SS
CEBPB			SS	MMSS	SS
CEBPD				MM	
E2F6					MM
EBF1	MMSS				
EGR1	MMM	MM			MM
ELF1	MM			MM	MM
ETS1	MM				MM
FAM48A (SPT20)			SS		
FOS (c-Fos)	SSS		SS		SSS
FOSL1		MM			MM
FOSL2				MM	
FOXA1				MMMM	
FOXA2				MM	
GABPA (GABP)	MM	MM	MM	MM	MM
GATA2					MM
HEY1				MM	
HNF4A				MM	
HNF4G				MM	
IRF3	SS		SS	SS	
IRF4	MM				
JUN (c-Jun)		SS	SS	SS	SS
JUND	SS	MMSS	SS	MMSS	SS
MAFF				SS	SS
MAFK				SSSS	SS
MAX	SS		SSSS	SS	MMSSSS
MEF2A	MM				MM
MEF2C	MM				
MTA3	MM				
MXI1	SS		SS		SS
MYC (c-Myc)		SS	SS		SSSS
NANOG		MM			
NFATC1	MM				
NFE2	SS				SS
NFIC				MM	
NFYA					SS

	GM12878	H1-hESC	HeLa-S3	HepG2	K562
NFYB					SS
NRF1	SS	SS	SS	SS	SS
PAX5	MMMM				
PBX3	MM				
PML	MM				MM
POU2F2	MMM				
POU5F1		MM			
PRDM1			SS		
REST (NRSF)	MMMM	MM	MM	MMMM	MM
RFX5	SS	SS	SS	SS	SS
RUNX3	MM				
RXRA	MM	MM		MM	
SIN3A		MM		MM	MM
SIX5	MM	MM			MMMM
SP1	MM	MM		MM	MM
SP2		MM		MM	MM
SP4		MM			
SPI1 (PU.1)	MMM				MM
SRF	MMMM	MM		MM	MM
STAT1	SS				
STAT3	SS		SS		
STAT5A	MM				
TAL1					SS
TCF12	MM	MM		MM	
TCF3	MM				
TEAD4					MM
THAP1					MM
USF1	MM	MM		MM	MM
USF2	SS	SS	SS	SS	SS
YY1	MM	MM		MM	MMMM
ZBTB33	MM			MMMM	MM
ZBTB7A				MM	MM
ZEB1	MM				
ZNF143	SS	SS	SS		SS
cohesin complex					
CTCF	SS	MM		MM	MMSS
CTCFL					MM
RAD21	MMSS	MMSS	SS	MMSS	MMSS
SMC3	SS		SS	SS	SS
chromatin remodeler					
BRCA1	SS	SS	SS	SS	
CHD2	SS		SS	SS	SS
EP300 (p300)	MMSSSSSS	MM	SS	MMSS	SSSS
HDAC2		MM		MM	MM
KAT2A (GCN5)			SS		
MBD4				MM	
SMARCA4 (BRG1)			SSS		SS
SMARCB1 (INI1)			SSS		SS
SMARCC1 (BAF155)			SSS		

	GM12878	H1-hESC	HeLa-S3	HepG2	K562
SMARCC2 (BAF170)			SSS		
WRNIP1 (WHIP)	SS				
XRCC4					SS
preinitiation complex					
GTF2F1			SS		SS
TAF1	MM	MM	MM	MM	MM
TAF7		MM			MM
TBP	SS	SS	SS	SS	SS
polymerase					
Pol II-S2P	SS	SS	SS		SS
Pol II-S5P	MMSSSSSSS	MM	MMSSS	MMSS	MMSSSS
control					
input	MMMMMMMSSSS	MS	MSSSS	MMMMMMS	MMMMSSSSS
histone mark					
H3K27ac	BB	BB	BB	BB	BB
H3K4me1	BB	BB	BB	BB	BB
H3K4me3	BB	BB	BB	BB	BB

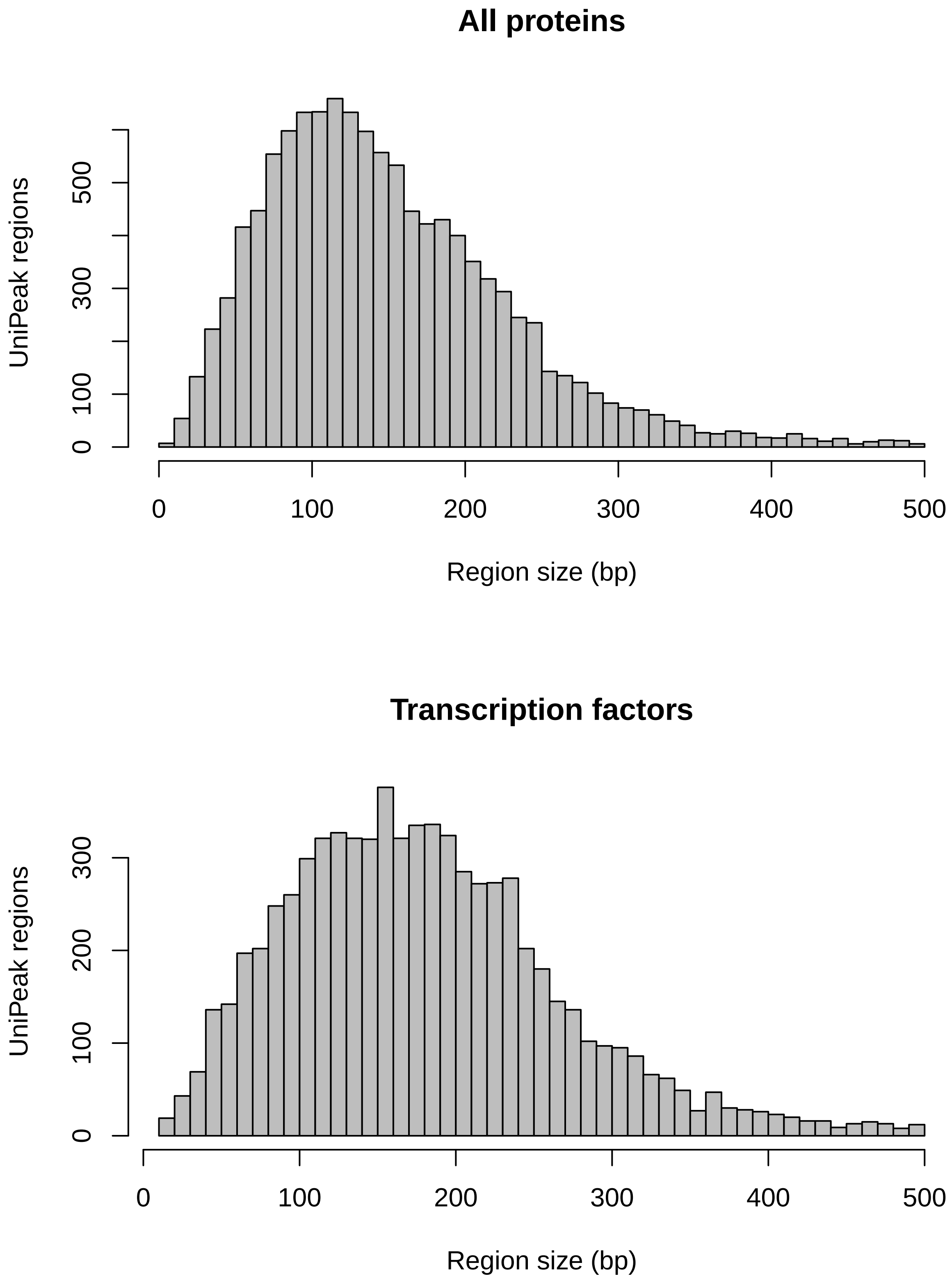
All proteins
UniPeak regions
0
100
300
500
0
100
200
300
400
500
Region size (bp)
Transcription factors
UniPeak regions
0
100
200
300
0
100
200
300
400
500
Region size (bp)

Figure S3

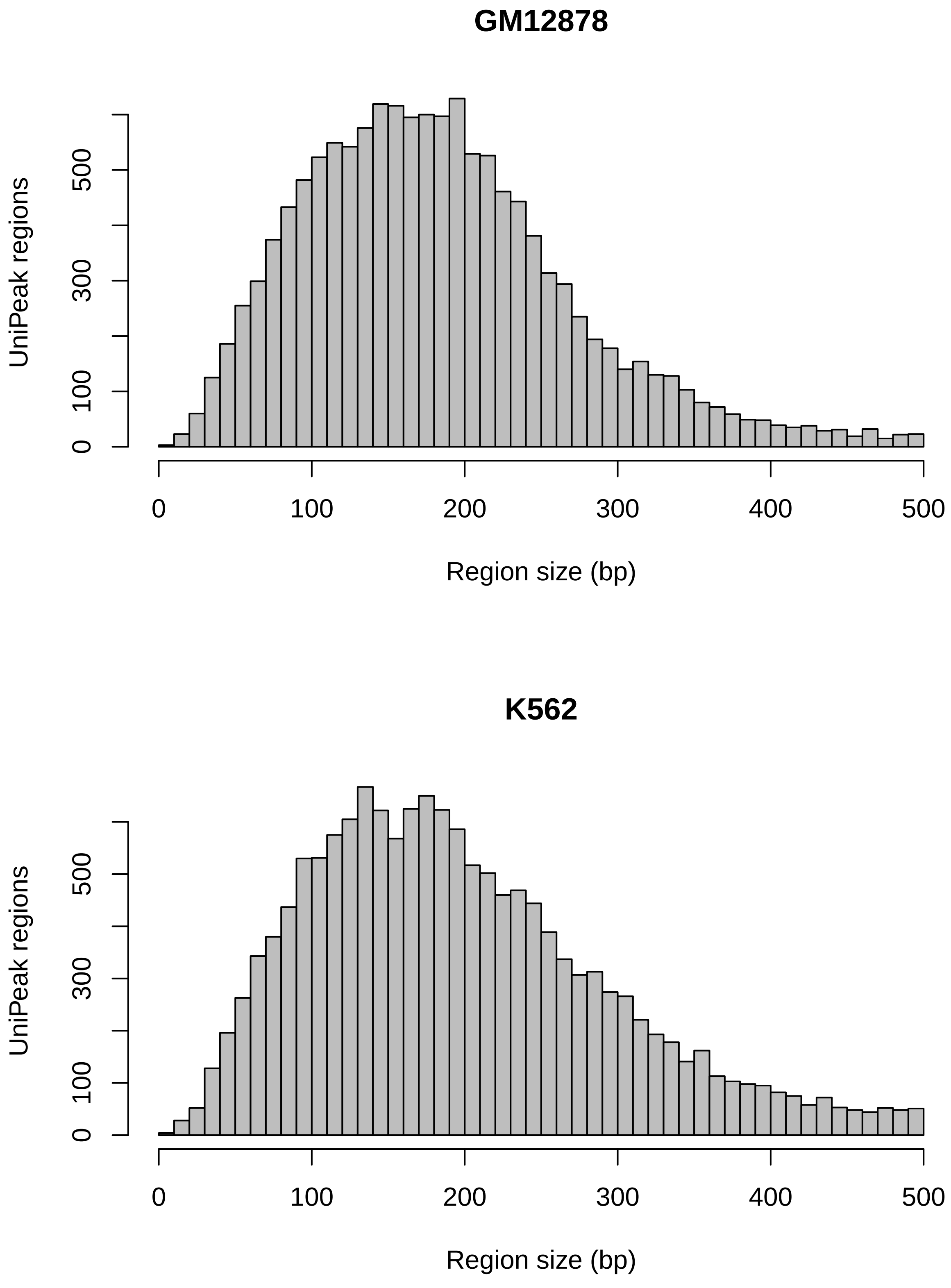
GM12878
UniPeak regions
0
100
300
500
0
100
200
300
400
500
Region size (bp)
K562
UniPeak regions
0
100
300
500
0
100
200
300
400
500
Region size (bp)

Figure S3

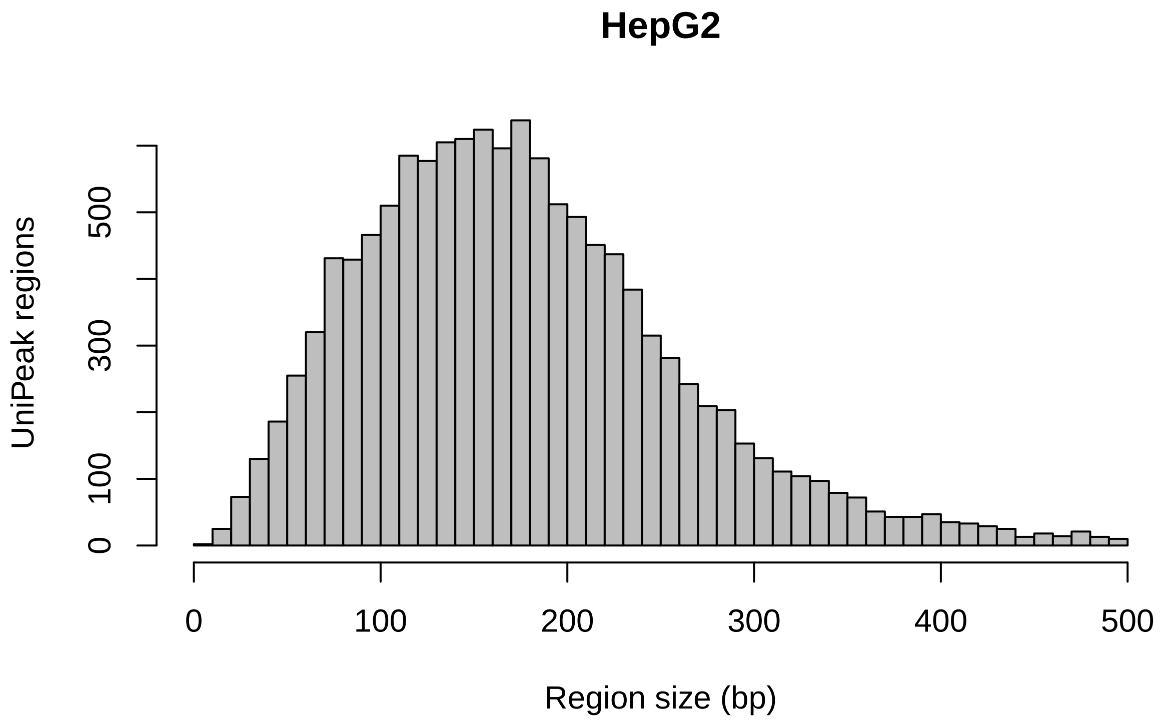
HepG2
UniPeak regions
0
100
300
500
0
100
200
300
400
500
Region size (bp)

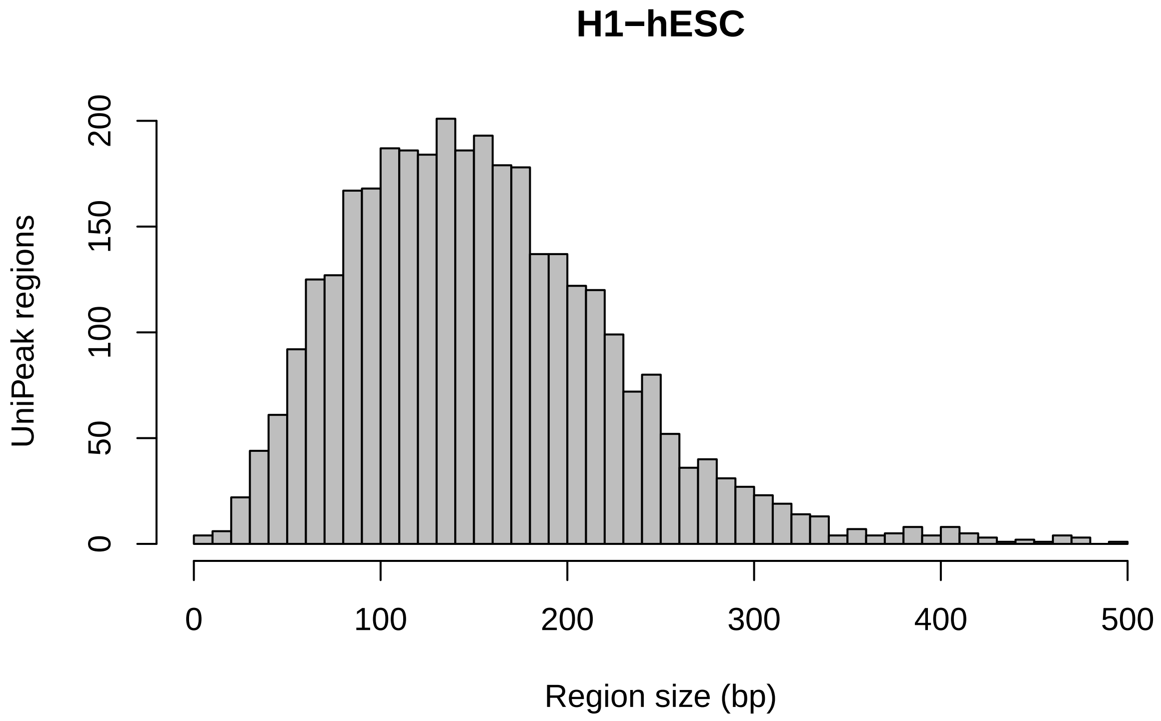
H1−hESC
UniPeak regions
0
50
100
150
200
0
100
200
300
400
500
Region size (bp)

Figure S3

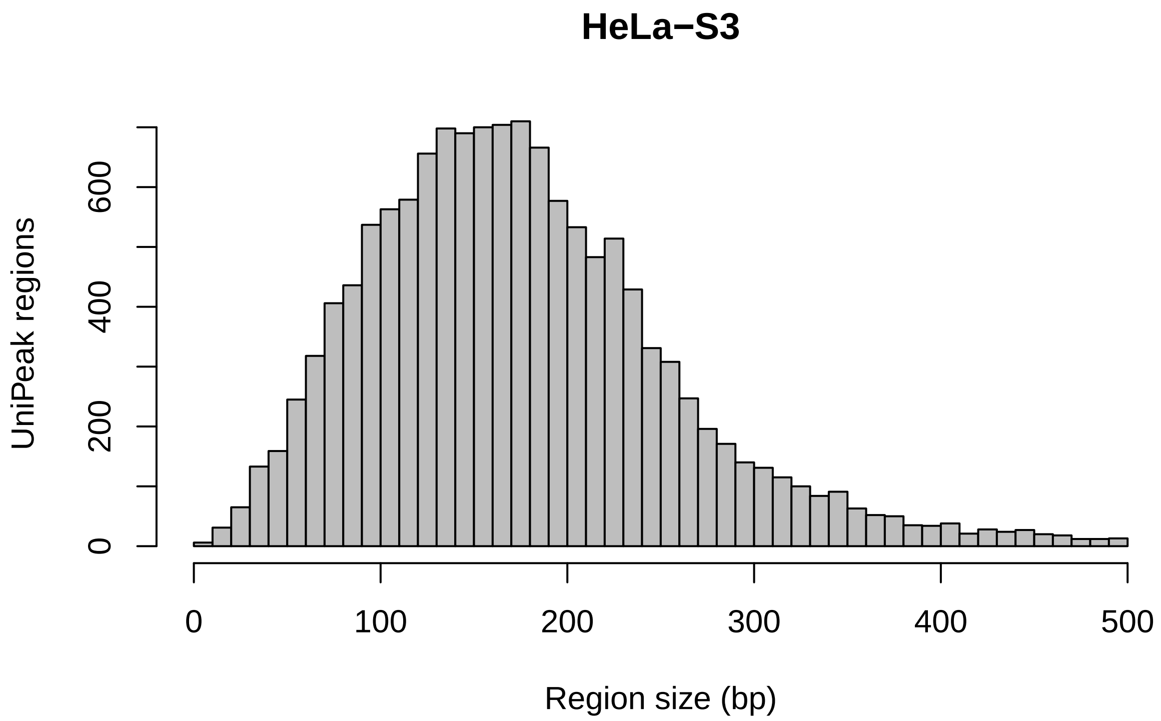

Figure S3: Distribution of `UniPeak` region sizes in all analyses.

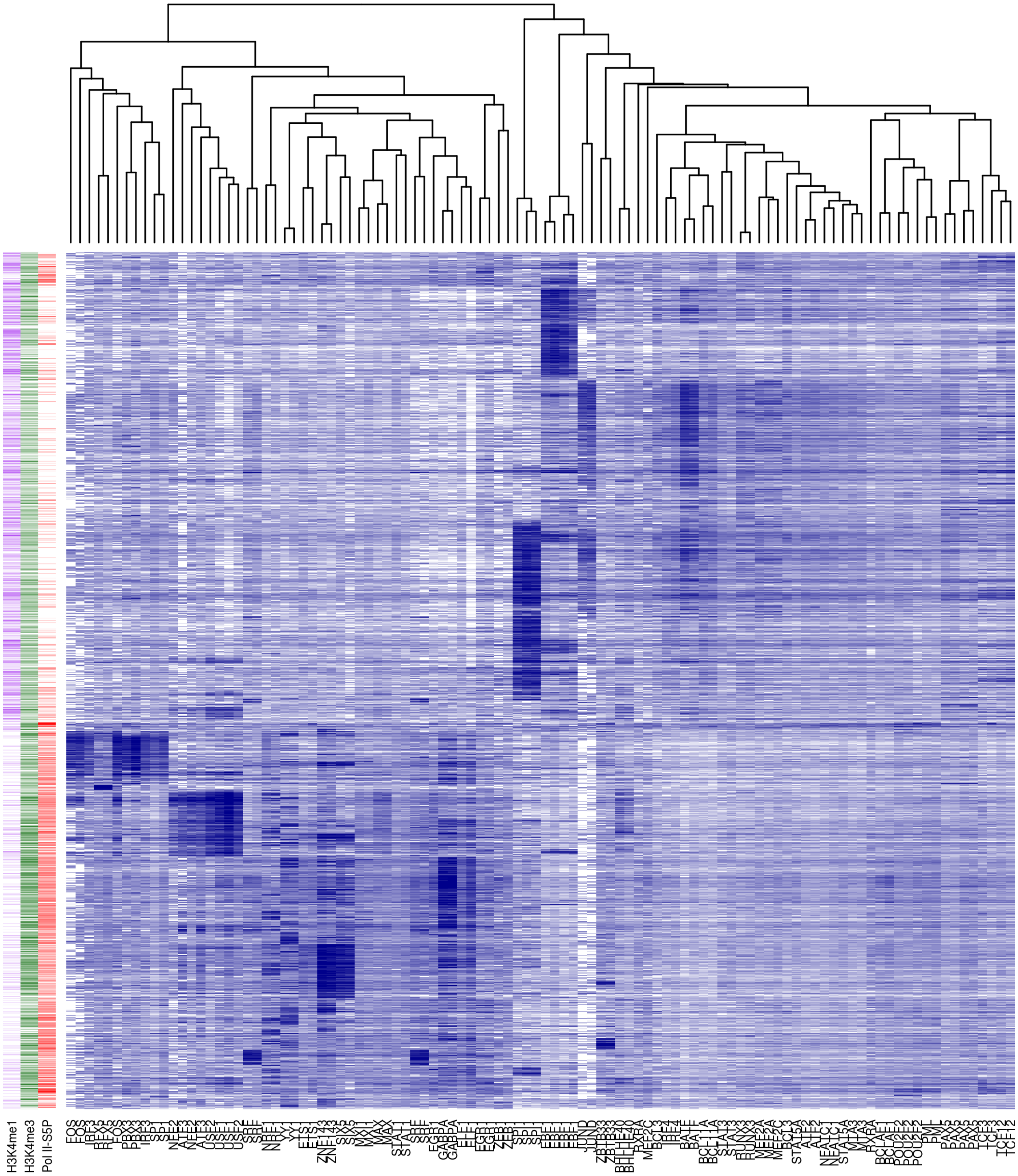

Figure S4

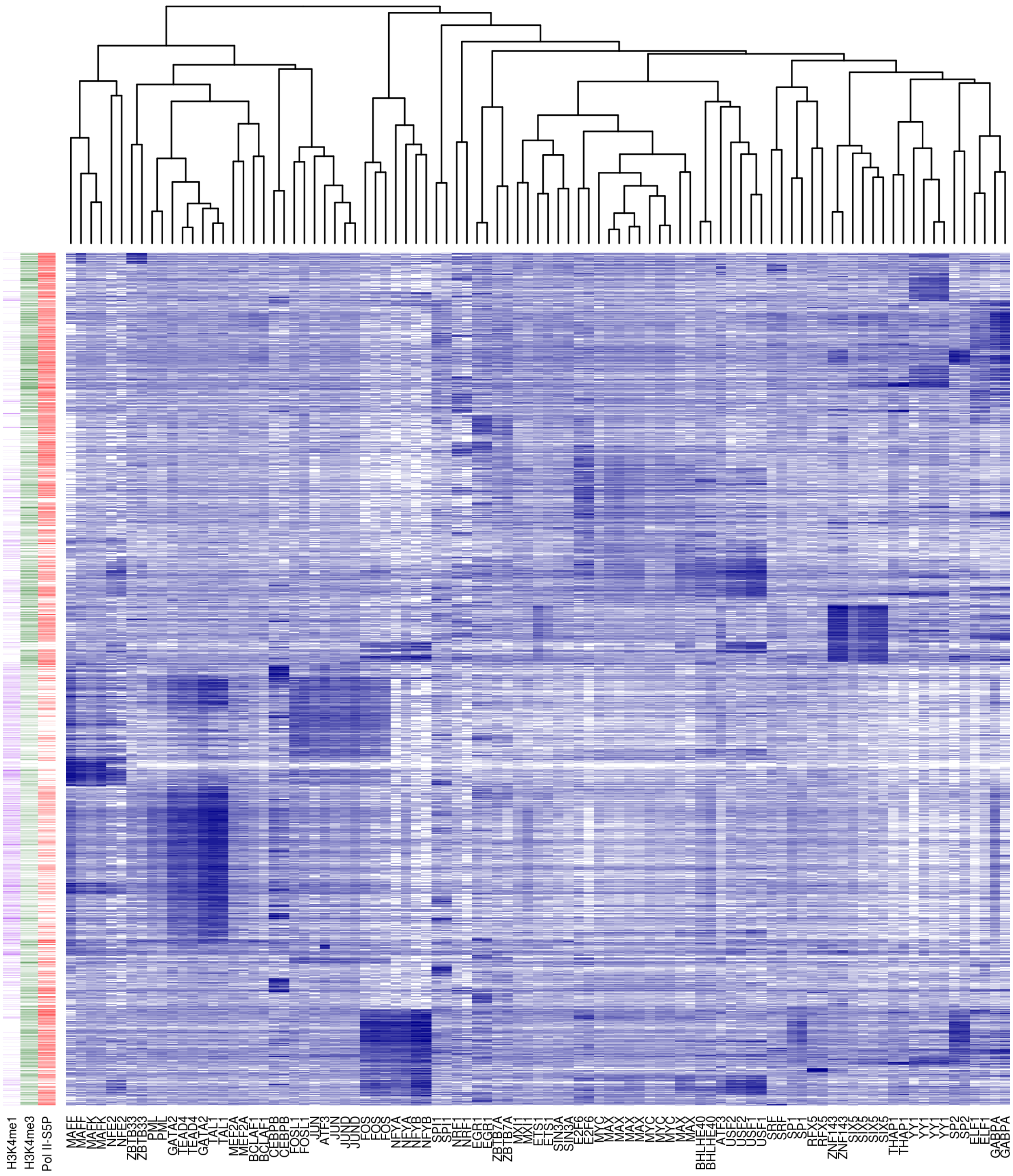
H3K4me1
H3K4me3
Pol II-S5P
MAFF
MAFF
MAFK
MAFK
NFE2
NFE2
ZBTB33
ZBTB33
PML
PML
GATA2
TEAD4
TEAD4
GATA2
TAL1
TAL1
MEF2A
MEF2A
BCLAF1
BCLAF1
CEBPB
CEBPB
FOSL1
FOSL1
JUN
ATF3
JUN
JUND
JUND
FOS
FOS
FOS
NFYA
NFYA
NFYB
NFYB
SPI1
SPI1
NRF1
NRF1
EGR1
EGR1
ZBTB7A
ZBTB7A
MXI1
MXI1
ETS1
ETS1
SIN3A
SIN3A
E2F6
E2F6
MYC
MAX
MAX
MAX
MAX
MYC
MYC
MYC
MAX
MAX
BHLHE40
BHLHE40
ATF3
USF2
USF2
USF1
USF1
SRF
SRF
SP1
SP1
RFX5
RFX5
ZNF143
ZNF143
SIX5
SIX5
SIX5
SIX5
THAP1
THAP1
YY1
YY1
YY1
YY1
SP2
SP2
ELF1
ELF1
GABPA
GABPA

Figure S4

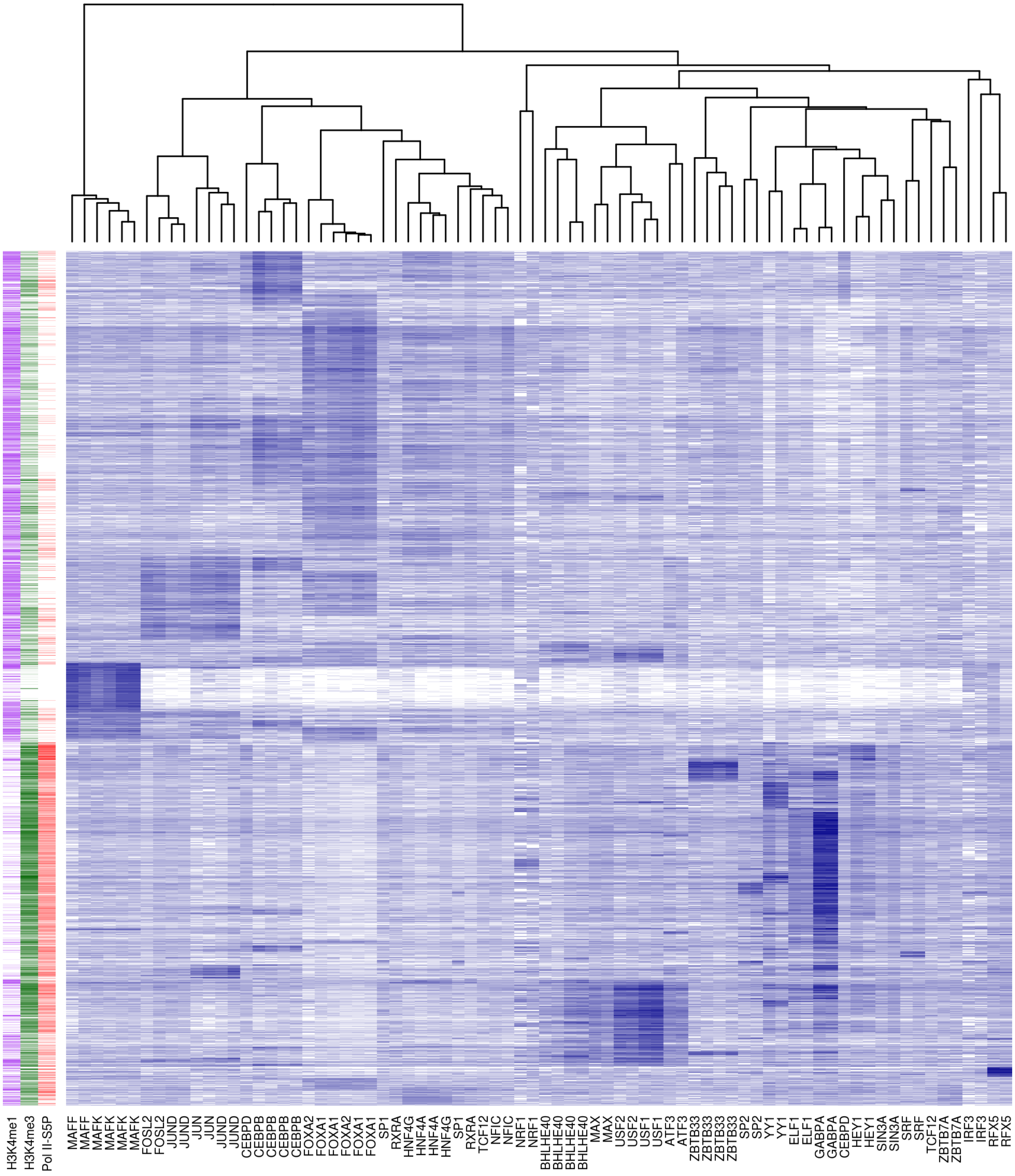
H3K4me1
H3K4me3
Pol II-S5P
MAFF
MAFF
MAFK
MAFK
MAFK
MAFK
FOSL2
FOSL2
JUND
JUND
JUN
JUN
JUND
JUND
CEBPD
CEBPB
CEBPB
CEBPB
CEBPB
FOXA2
FOXA1
FOXA2
FOXA1
FOXA1
FOXA1
SP1
RXRA
HNF4G
HNF4A
HNF4A
HNF4G
SP1
RXRA
TCF12
NFIC
NFIC
NRF1
NRF1
BHLHE40
BHLHE40
BHLHE40
BHLHE40
MAX
MAX
USF2
USF2
USF1
USF1
ATF3
ATF3
ZBTB33
ZBTB33
ZBTB33
ZBTB33
SP2
SP2
YY1
YY1
ELF1
ELF1
GABPA
GABPA
CEBPD
HEY1
HEY1
SIN3A
SIN3A
SRF
SRF
TCF12
ZBTB7A
ZBTB7A
IRF3
IRF3
RFX5
RFX5

Figure S4

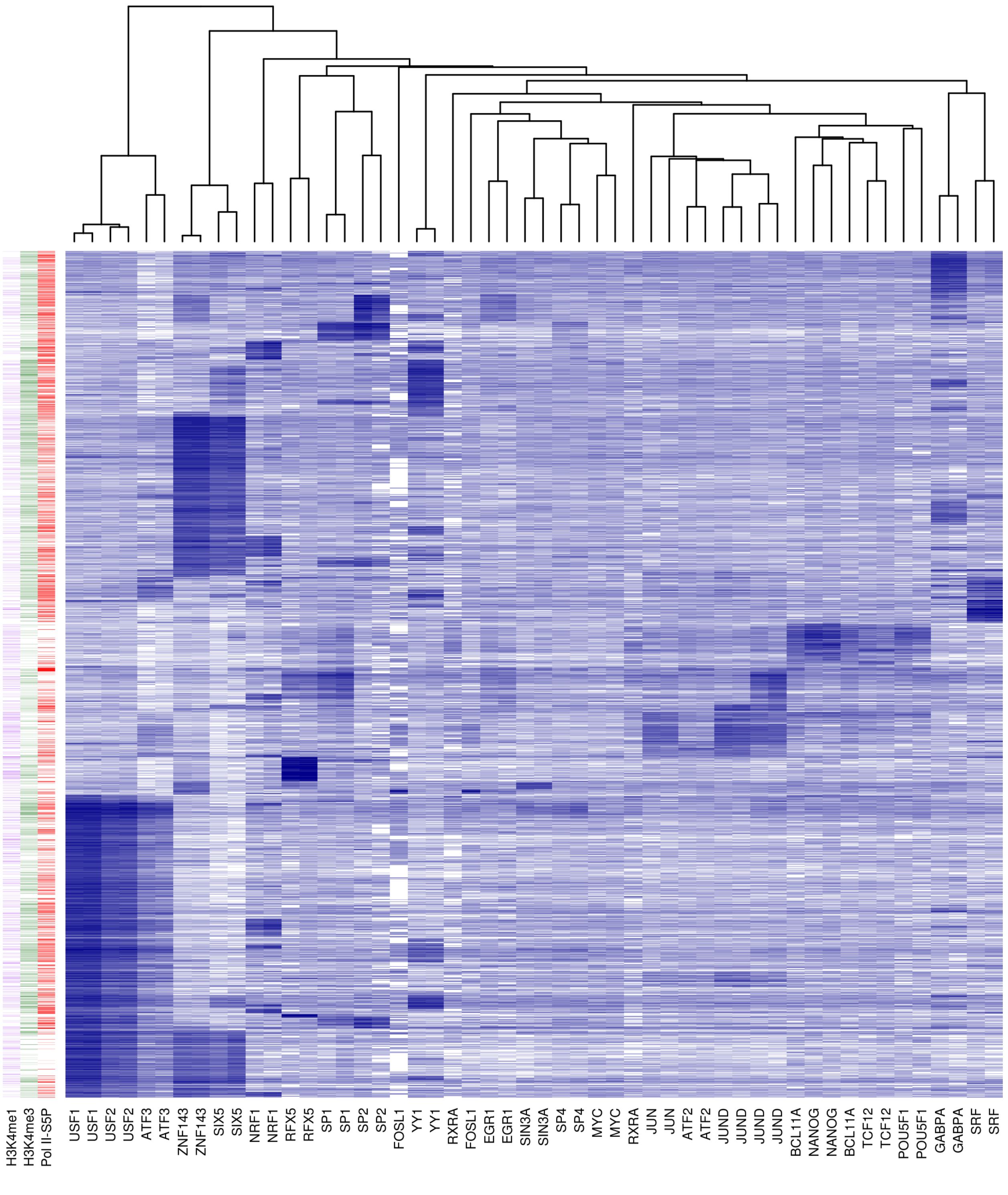
H3K4me1
H3K4me3
Pol II-S5P
USF1
USF1
USF2
USF2
ATF3
ATF3
ZNF143
ZNF143
SIX5
SIX5
NRF1
NRF1
RFX5
RFX5
SP1
SP1
SP2
SP2
FOSL1
YY1
YY1
RXRA
FOSL1
EGR1
EGR1
SIN3A
SIN3A
SP4
SP4
MYC
MYC
RXRA
JUN
JUN
ATF2
ATF2
JUND
JUND
JUND
JUND
BCL11A
NANOG
NANOG
BCL11A
TCF12
TCF12
POU5F1
POU5F1
GABPA
GABPA
SRF
SRF

Figure S4

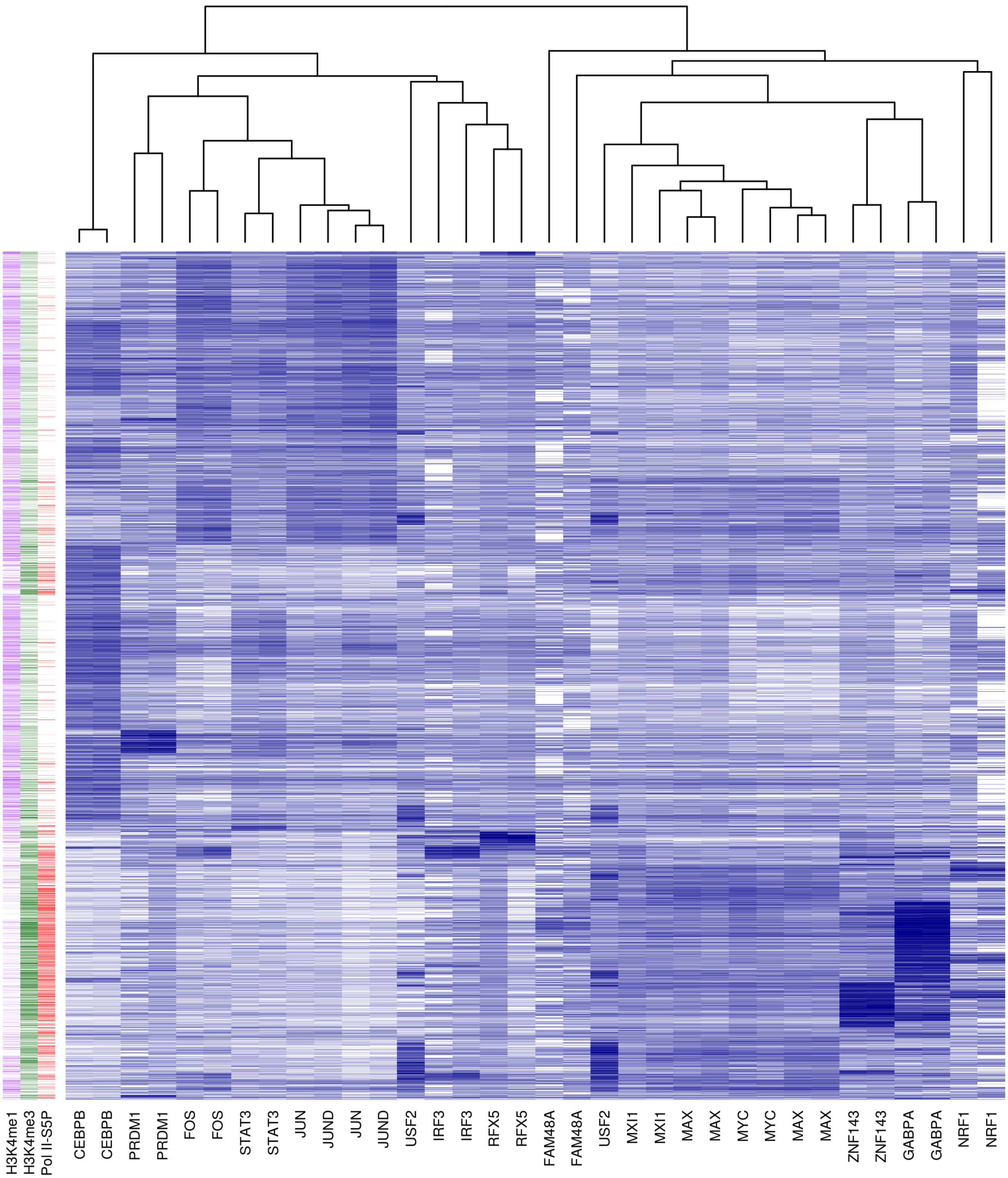

Figure S4: Clustered heatmaps for cell-specific analyses. S4a: GM12878. S4b: K562. S4c: HepG2. S4d: H1-hESC. S4e: HeLa-S3.

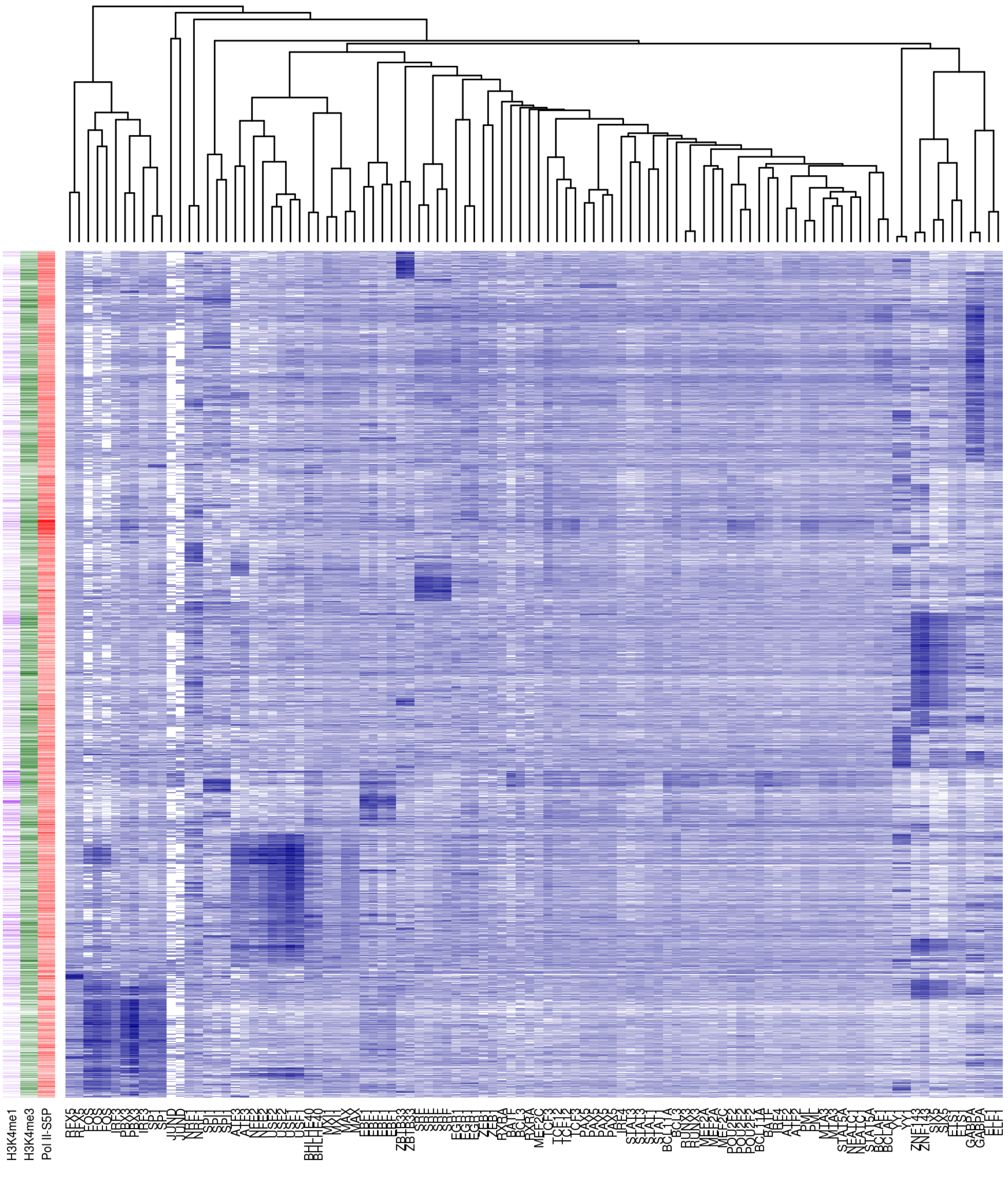

Figure S5

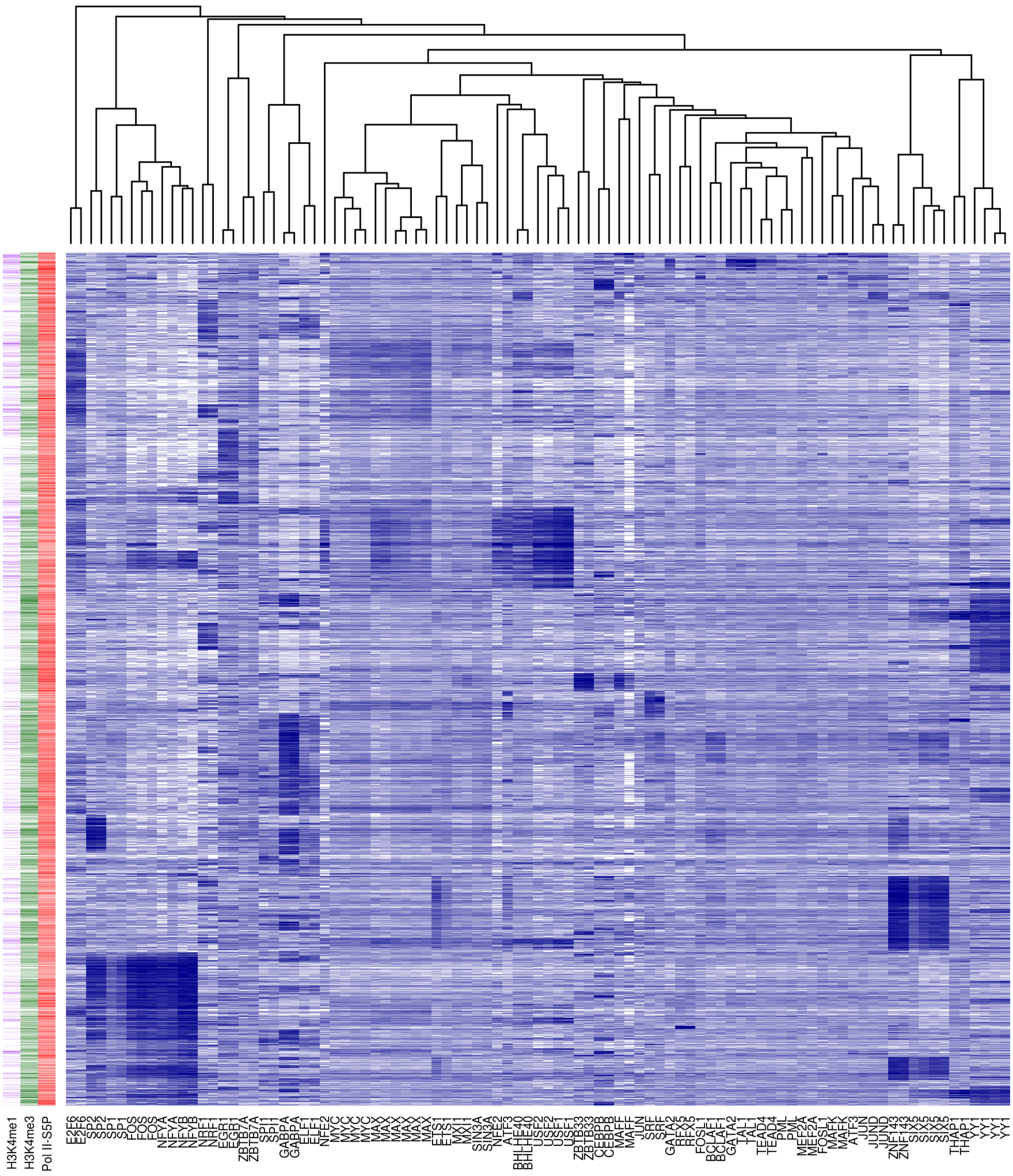
H3K4me1
H3K4me3
Pol II-S5P
E2F6
E2F6
SP2
SP2
SP1
SP1
FOS
FOS
FOS
NFYA
NFYA
NFYB
NFYB
NRF1
NRF1
EGR1
EGR1
ZBTB7A
ZBTB7A
SPI1
SPI1
GABPA
GABPA
ELF1
ELF1
NFE2
MYC
MYC
MYC
MYC
MAX
MAX
MAX
MAX
MAX
ETS1
ETS1
MXI1
MXI1
SIN3A
SIN3A
NFE2
ATF3
BHLHE40
BHLHE40
USF2
USF2
USF1
USF1
ZBTB33
ZBTB33
CEBPB
CEBPB
MAFF
MAFF
JUN
SRF
SRF
GATA2
RFX5
RFX5
FOSL1
BCLAF1
BCLAF1
GATA2
TAL1
TAL1
TEAD4
TEAD4
PML
PML
MEF2A
MEF2A
FOSL1
MAFK
MAFK
ATF3
JUN
JUND
JUND
ZNF143
ZNF143
SIX5
SIX5
SIX5
THAP1
THAP1
YY1
YY1
YY1
YY1

Figure S5

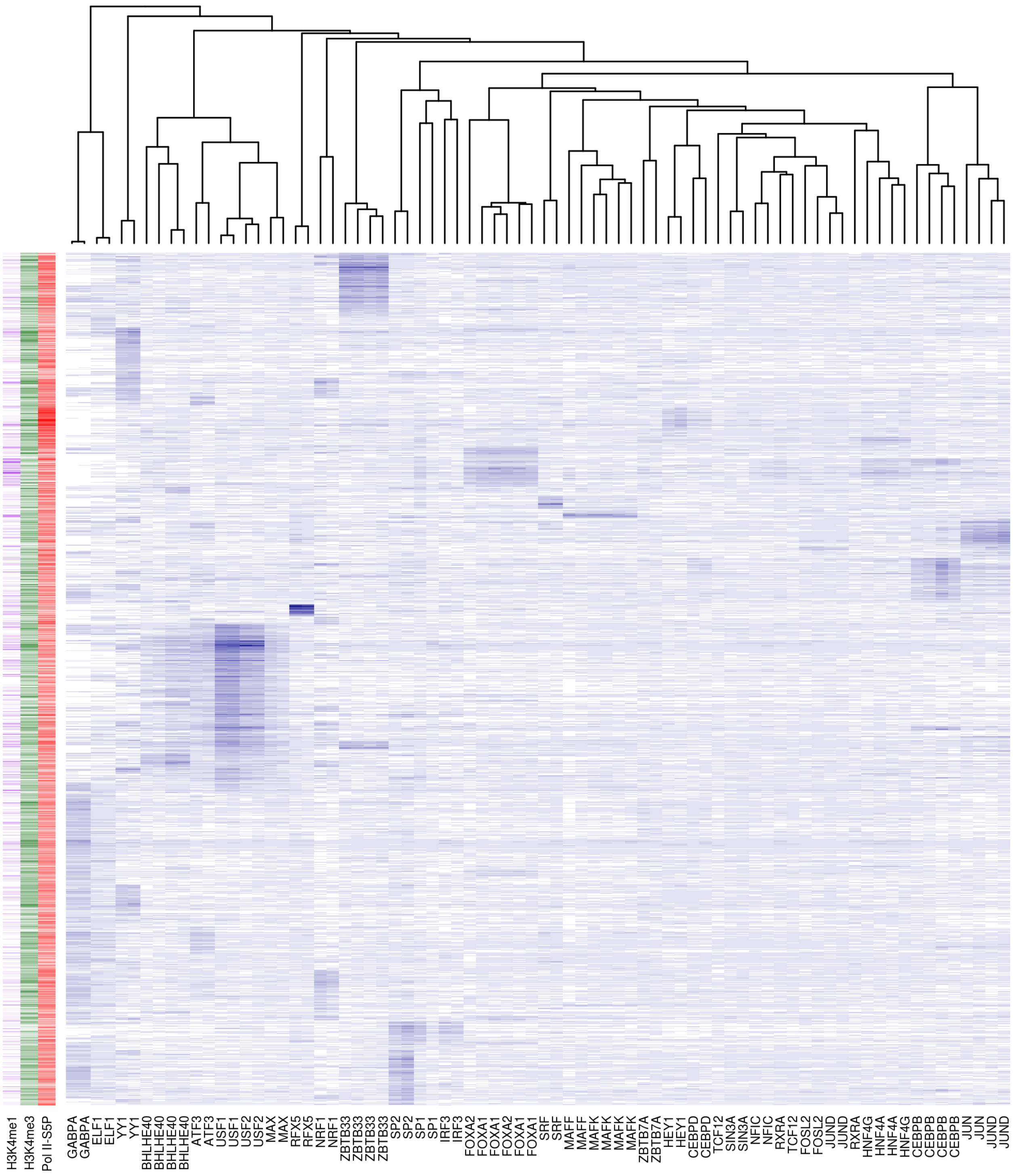
H3K4me1
H3K4me3
Pol II-S5P
GABPA
GABPA
ELF1
ELF1
YY1
YY1
BHLHE40
BHLHE40
BHLHE40
BHLHE40
ATF3
ATF3
USF1
USF1
USF2
USF2
MAX
MAX
RFX5
RFX5
NRF1
NRF1
ZBTB33
ZBTB33
ZBTB33
ZBTB33
SP2
SP2
SP1
SP1
IRF3
IRF3
FOXA2
FOXA1
FOXA1
FOXA2
FOXA1
FOXA1
SRF
SRF
MAFF
MAFF
MAFK
MAFK
MAFK
MAFK
ZBTB7A
ZBTB7A
HEY1
HEY1
CEBPD
CEBPD
TCF12
SIN3A
SIN3A
NFIC
NFIC
RXRA
TCF12
FOSL2
FOSL2
JUND
JUND
RXRA
HNF4G
HNF4A
HNF4A
HNF4G
CEBPB
CEBPB
CEBPB
CEBPB
JUN
JUN
JUND
JUND

Figure S5

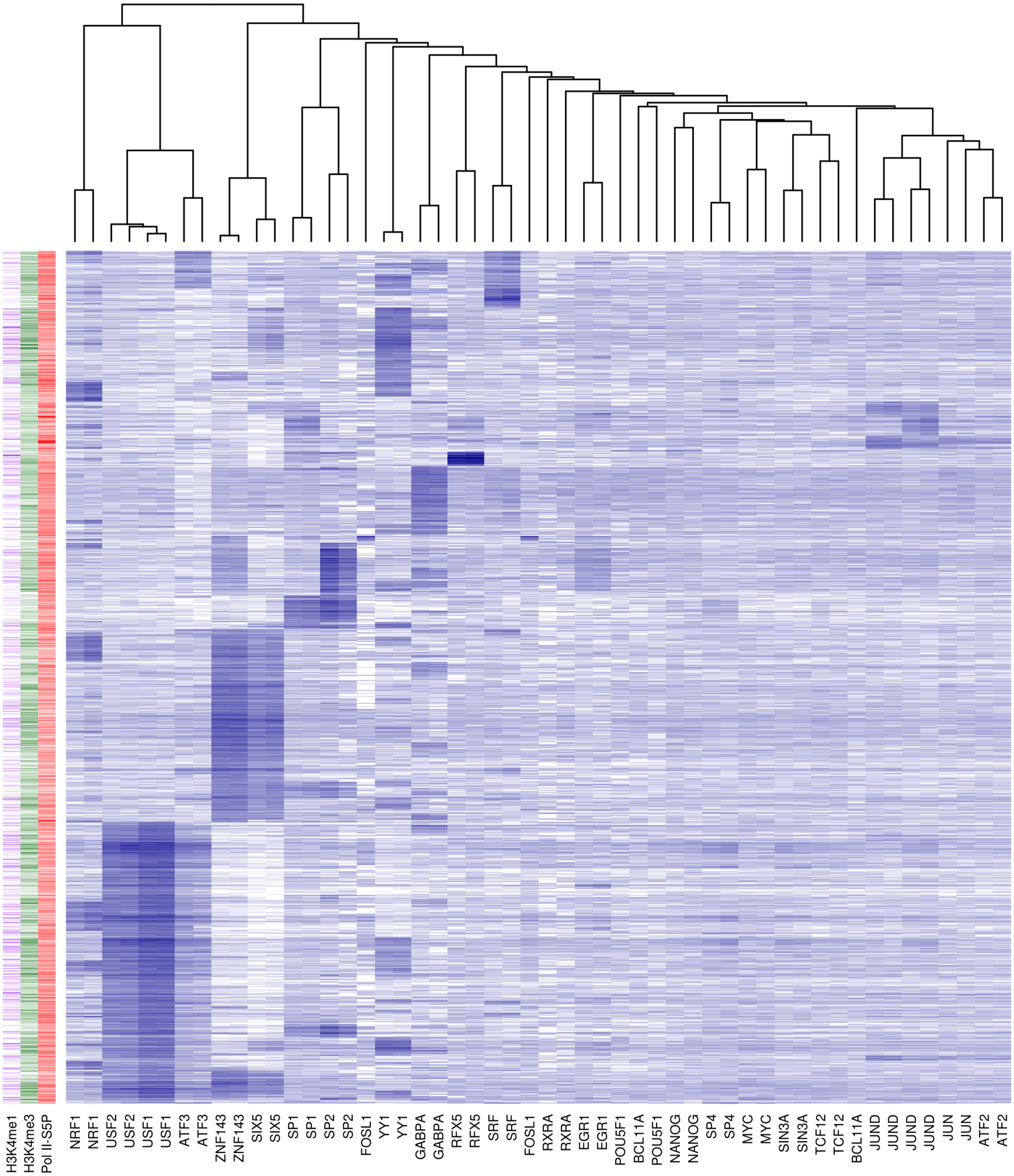
H3K4me1
H3K4me3
Pol II-S5P
NRF1
NRF1
USF2
USF2
USF1
USF1
ATF3
ATF3
ZNF143
ZNF143
SIX5
SIX5
SP1
SP1
SP2
SP2
FOSL1
YY1
YY1
GABPA
GABPA
RFX5
RFX5
SRF
SRF
FOSL1
RXRA
RXRA
EGR1
EGR1
POU5F1
BCL11A
POU5F1
NANOG
NANOG
SP4
SP4
MYC
MYC
SIN3A
SIN3A
TCF12
TCF12
BCL11A
JUND
JUND
JUND
JUND
JUN
JUN
ATF2
ATF2

Figure S5

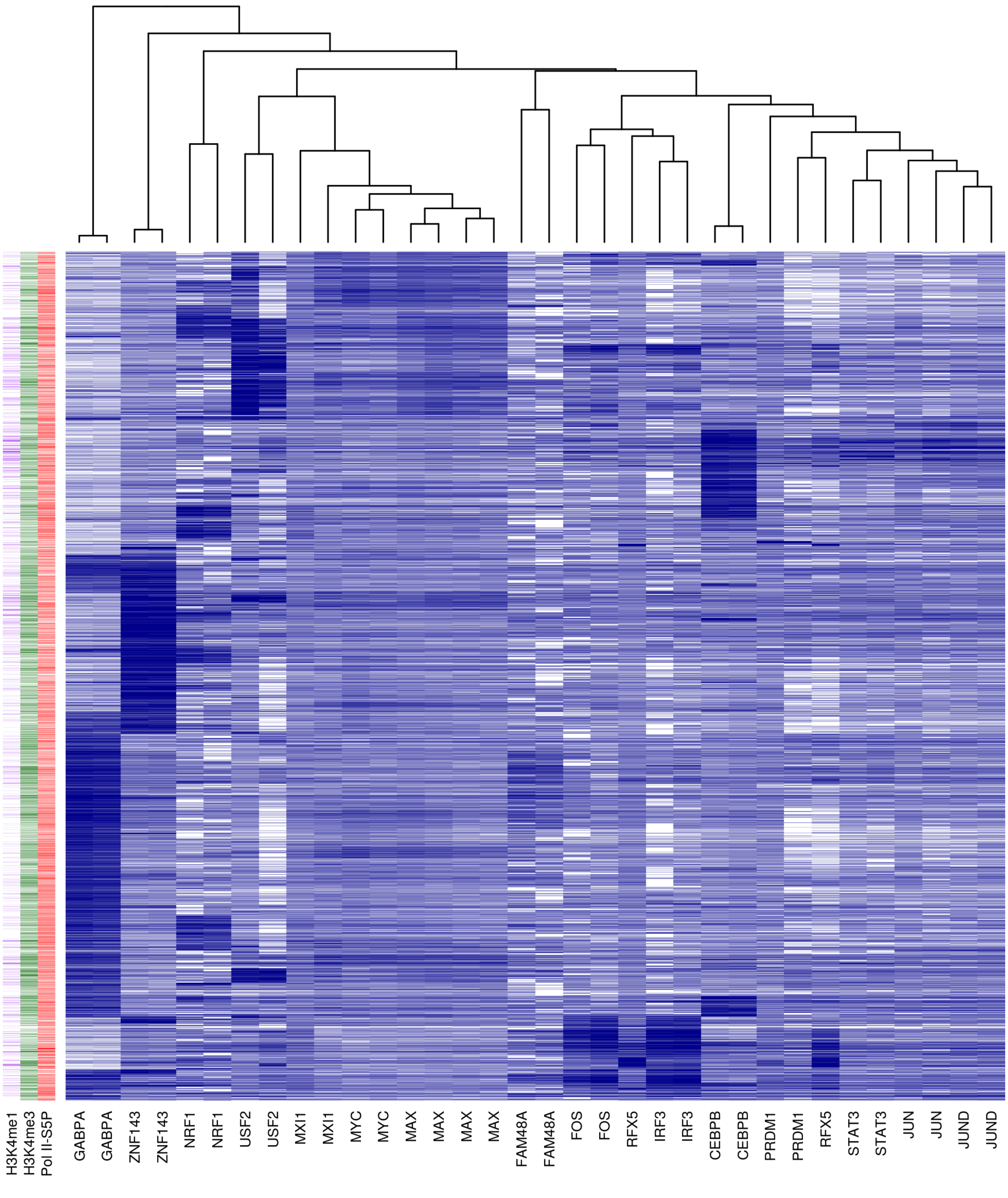

Figure S5: Clustered heatmaps for cell-specific analyses, consensus promoters only. S5a: GM12878. S5b: K562. S5c: HepG2. S5d: H1-hESC. S5e: HeLa-S3.

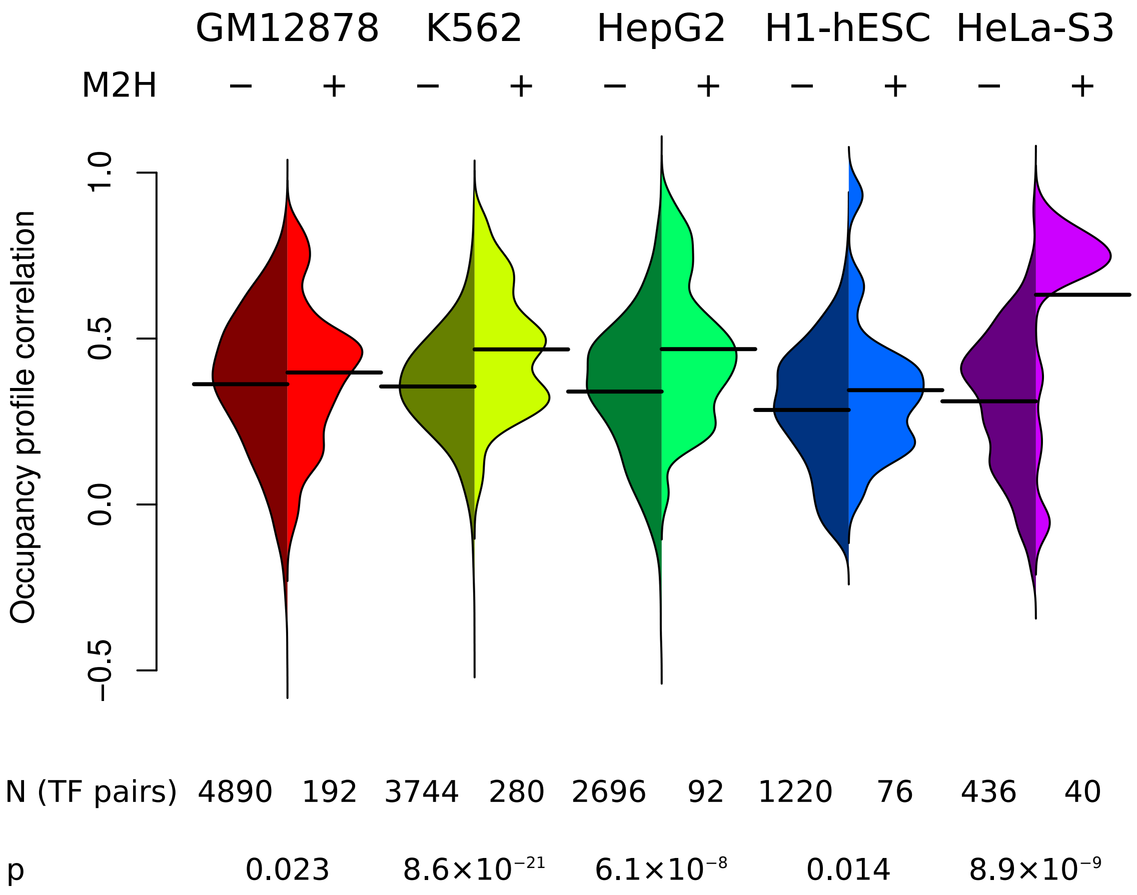

Figure S6: Occupancy profile correlations of interacting and non-interacting TFs. All pairwise correlations of occupancy profiles across all regions are plotted for each cell type, grouped by whether a mammalian two-hybrid (M2H) screen found the two TFs capable of directly binding each other. *p*-values are from the *t*-test.

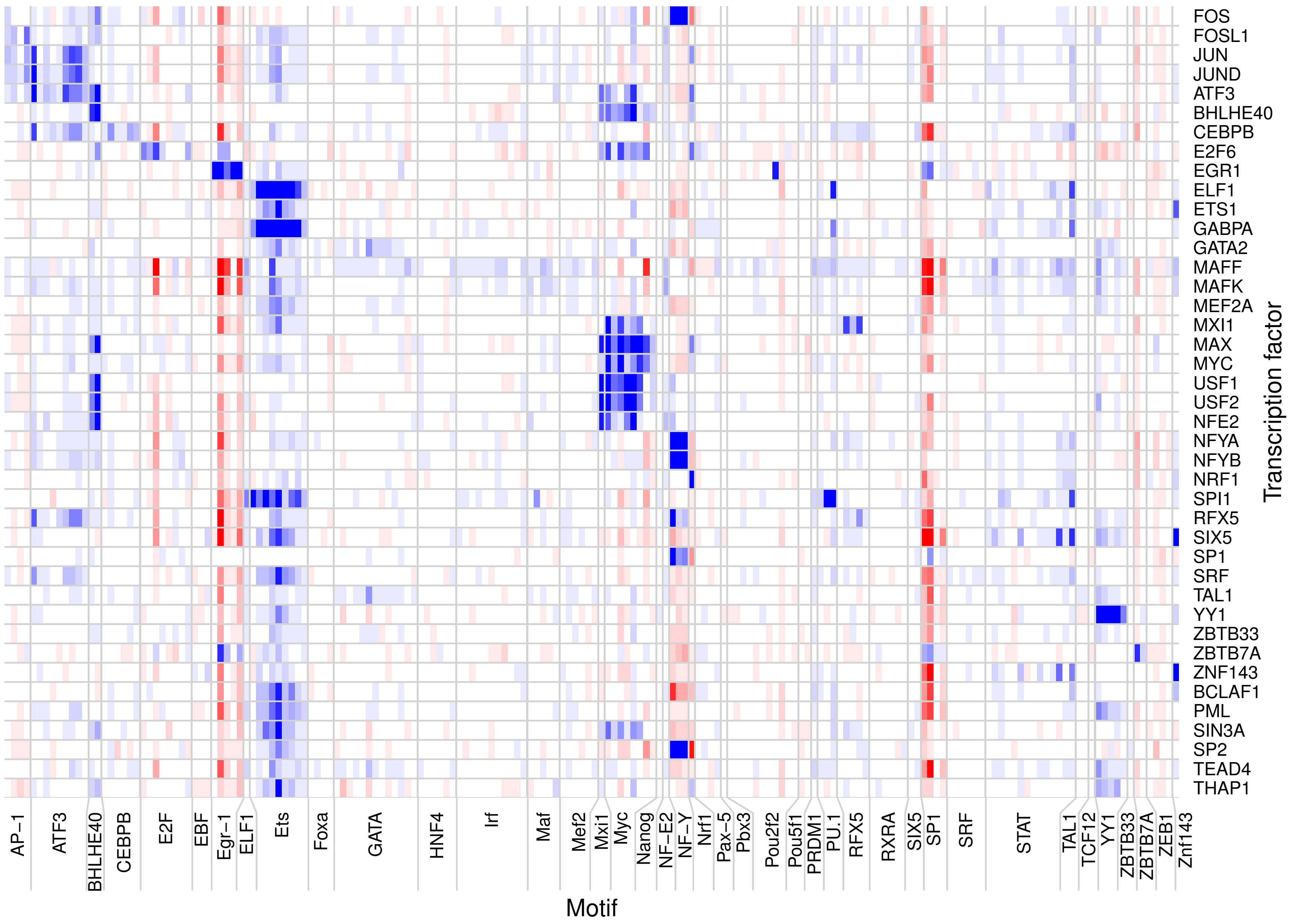

FOS
FOSL1
JUN
JUND
ATF3
BHLHE40
CEBPB
E2F6
EGR1
ELF1
ETS1
GABPA
GATA2
MAFF
MAFK
MEF2A
MXI1
MAX
MYC
USF1
USF2
NFE2
NFYA
NFYB
NRF1
SPI1
RFX5
SIX5
SP1
SRF
TAL1
YY1
ZBTB33
ZBTB7A
ZNF143
BCLAF1
PML
SIN3A
SP2
TEAD4
THAP1
Transcription factor
AP−1
ATF3
BHLHE40
CEBPB
E2F
EBF
Egr−1
ELF1
Ets
Foxa
GATA
HNF4
Irf
Maf
Mef2
Mxi1
Myc
Nanog
NF−E2
NF−Y
Nrf1
Pax−5
Pbx3
Pou2f2
Pou5f1
PRDM1
PU.1
RFX5
RXRA
SIX5
SP1
SRF
STAT
TAL1
TCF12
YY1
ZBTB33
ZBTB7A
ZEB1
Znf143
Motif

Figure S7

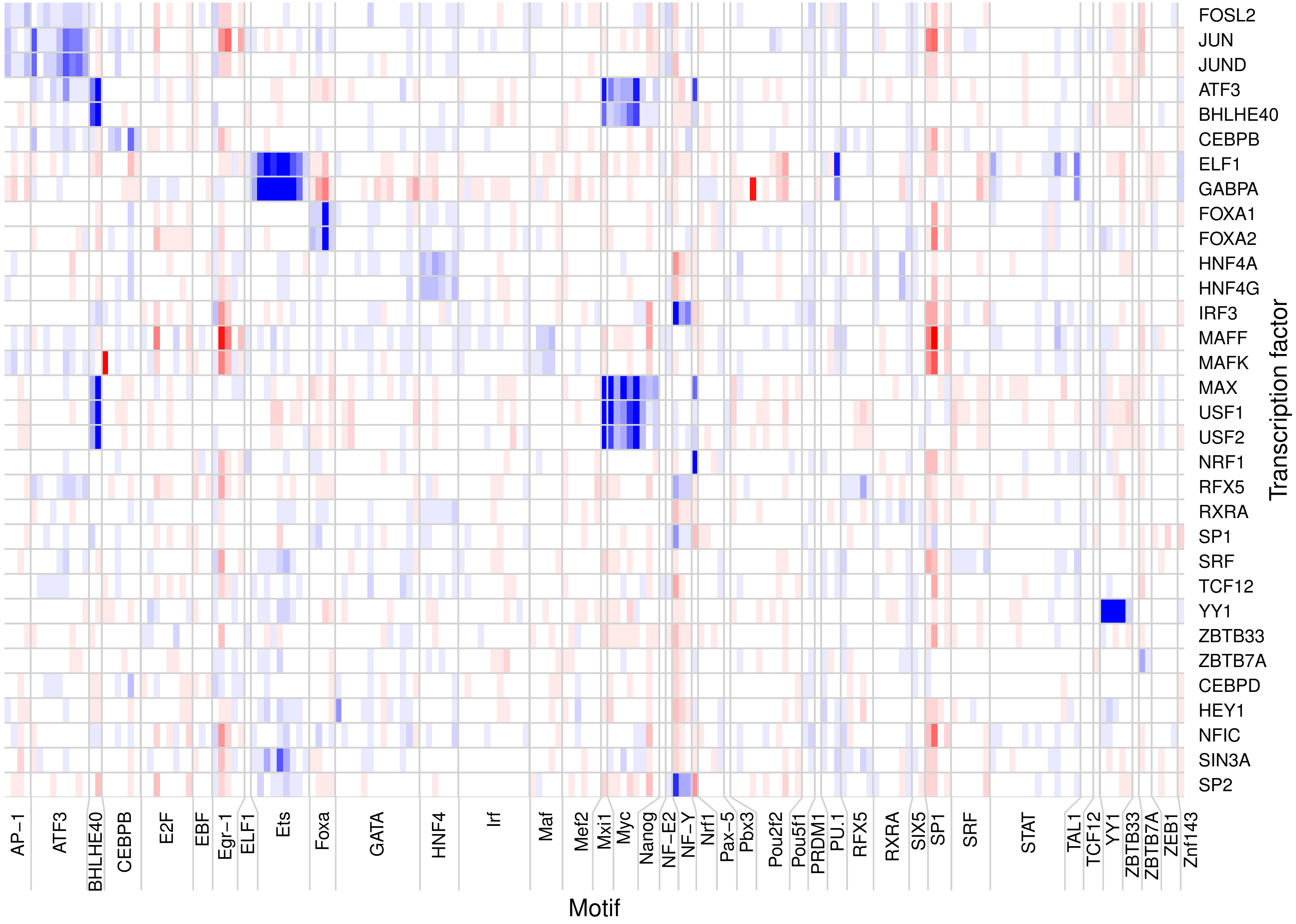
FOSL2
JUN
JUND
ATF3
BHLHE40
CEBPB
ELF1
GABPA
FOXA1
FOXA2
HNF4A
HNF4G
IRF3
MAFF
MAFK
MAX
USF1
USF2
NRF1
RFX5
RXRA
SP1
SRF
TCF12
YY1
ZBTB33
ZBTB7A
CEBPD
HEY1
NFIC
SIN3A
SP2
Transcription factor
AP−1
ATF3
BHLHE40
CEBPB
E2F
EBF
Egr−1
ELF1
Ets
Foxa
GATA
HNF4
Irf
Maf
Mef2
Mxi1
Myc
Nanog
NF−E2
NF−Y
Nrf1
Pax−5
Pbx3
Pou2f2
Pou5f1
PRDM1
PU.1
RFX5
RXRA
SIX5
SP1
SRF
STAT
TAL1
TCF12
YY1
ZBTB33
ZBTB7A
ZEB1
Znf143
Motif

Figure S7

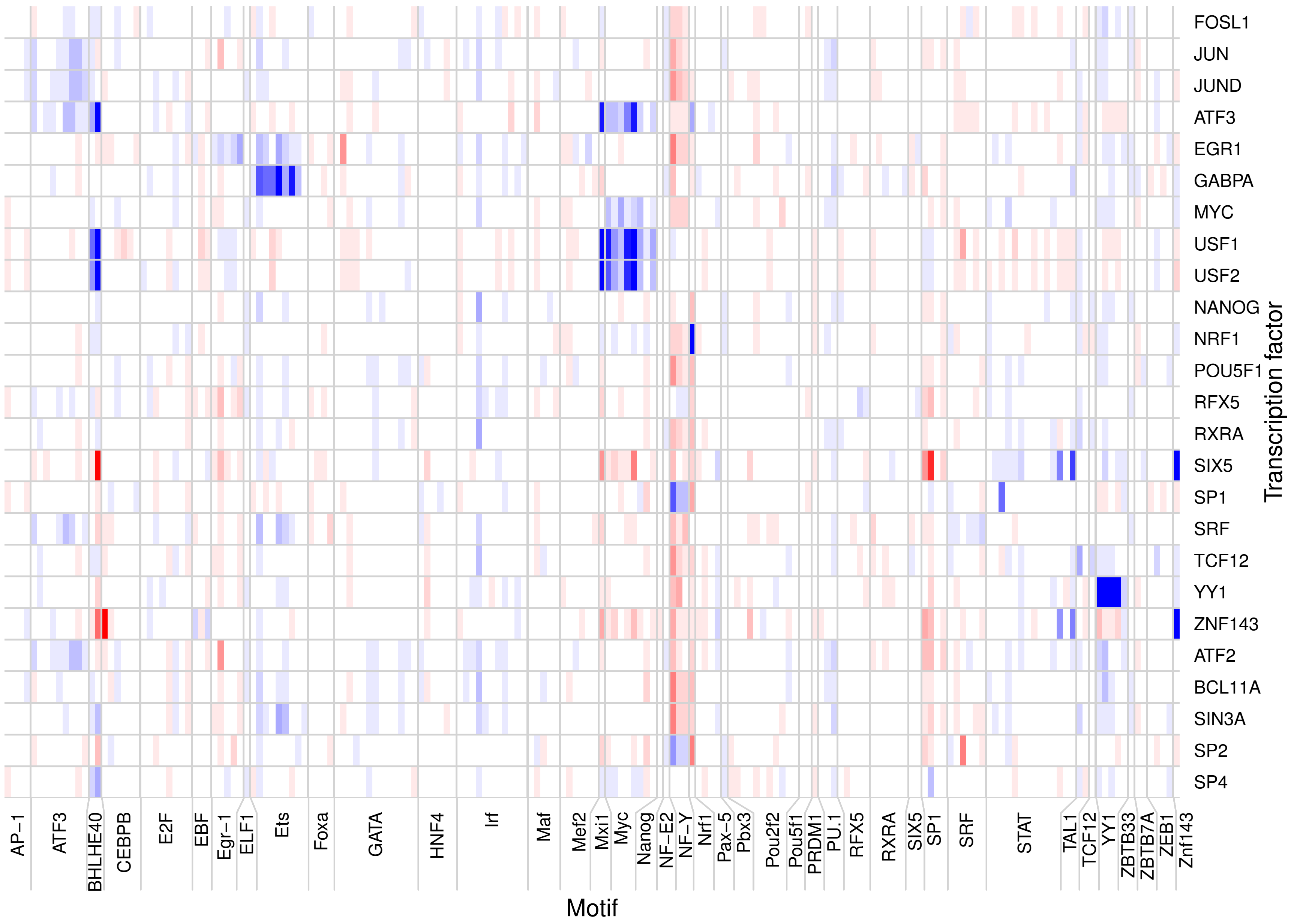
FOSL1
JUN
JUND
ATF3
EGR1
GABPA
MYC
USF1
USF2
NANOG
NRF1
POU5F1
RFX5
RXRA
SIX5
SP1
SRF
TCF12
YY1
ZNF143
ATF2
BCL11A
SIN3A
SP2
SP4
Transcription factor
AP−1
ATF3
BHLHE40
CEBPB
E2F
EBF
Egr−1
ELF1
Ets
Foxa
GATA
HNF4
Irf
Maf
Mef2
Mxi1
Myc
Nanog
NF−E2
NF−Y
Nrf1
Pax−5
Pbx3
Pou2f2
Pou5f1
PRDM1
PU.1
RFX5
RXRA
SIX5
SP1
SRF
STAT
TAL1
TCF12
YY1
ZBTB33
ZBTB7A
ZEB1
Znf143
Motif

Figure S7

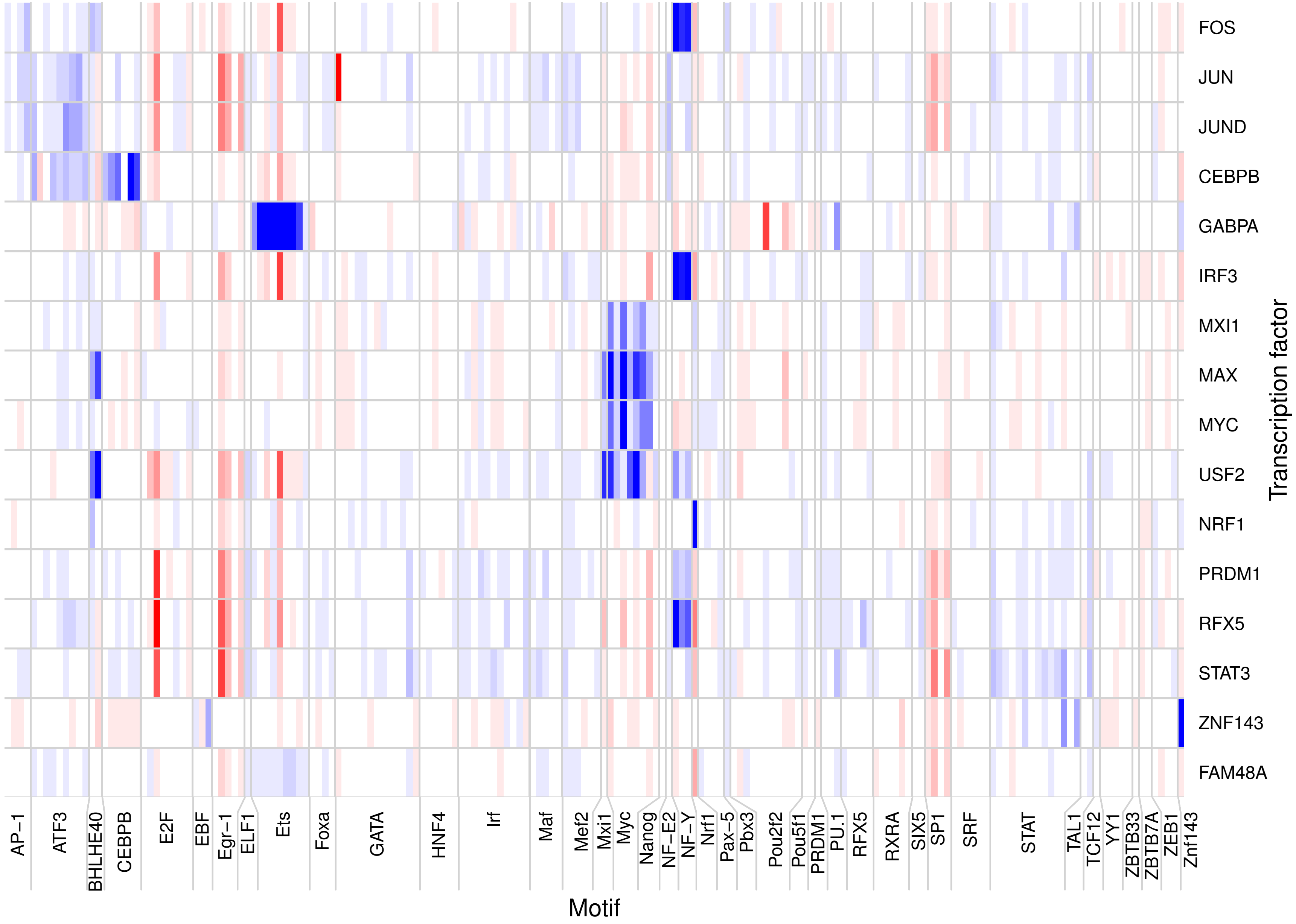

Figure S7: Sequence motifs vs. TF occupancy, by cell. S7a: K562. S7b: HepG2. S7c: H1-hESC. S7d: HeLa-S3.

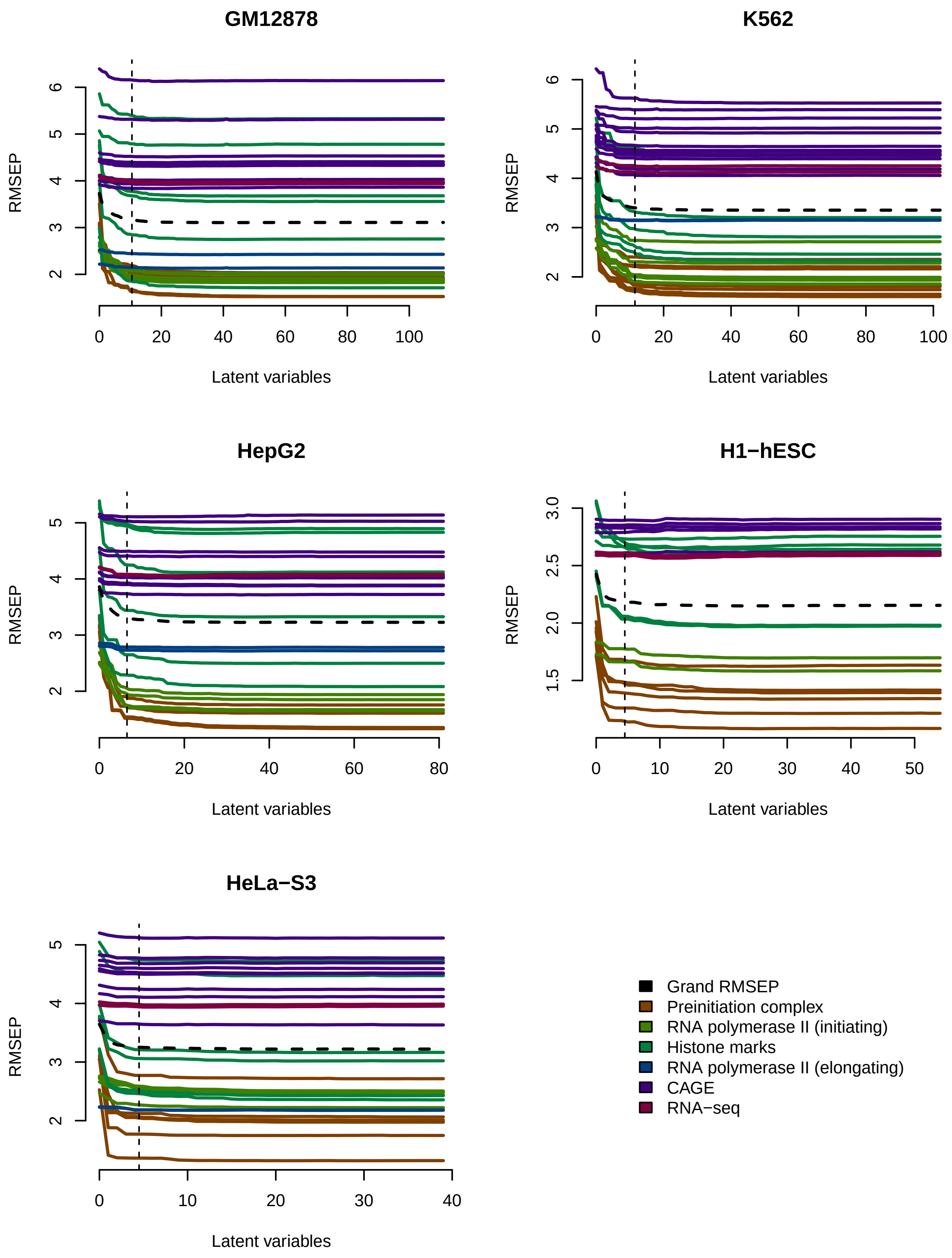

Figure S8: Root-mean-square error of prediction for partial least-squares regression analyses.

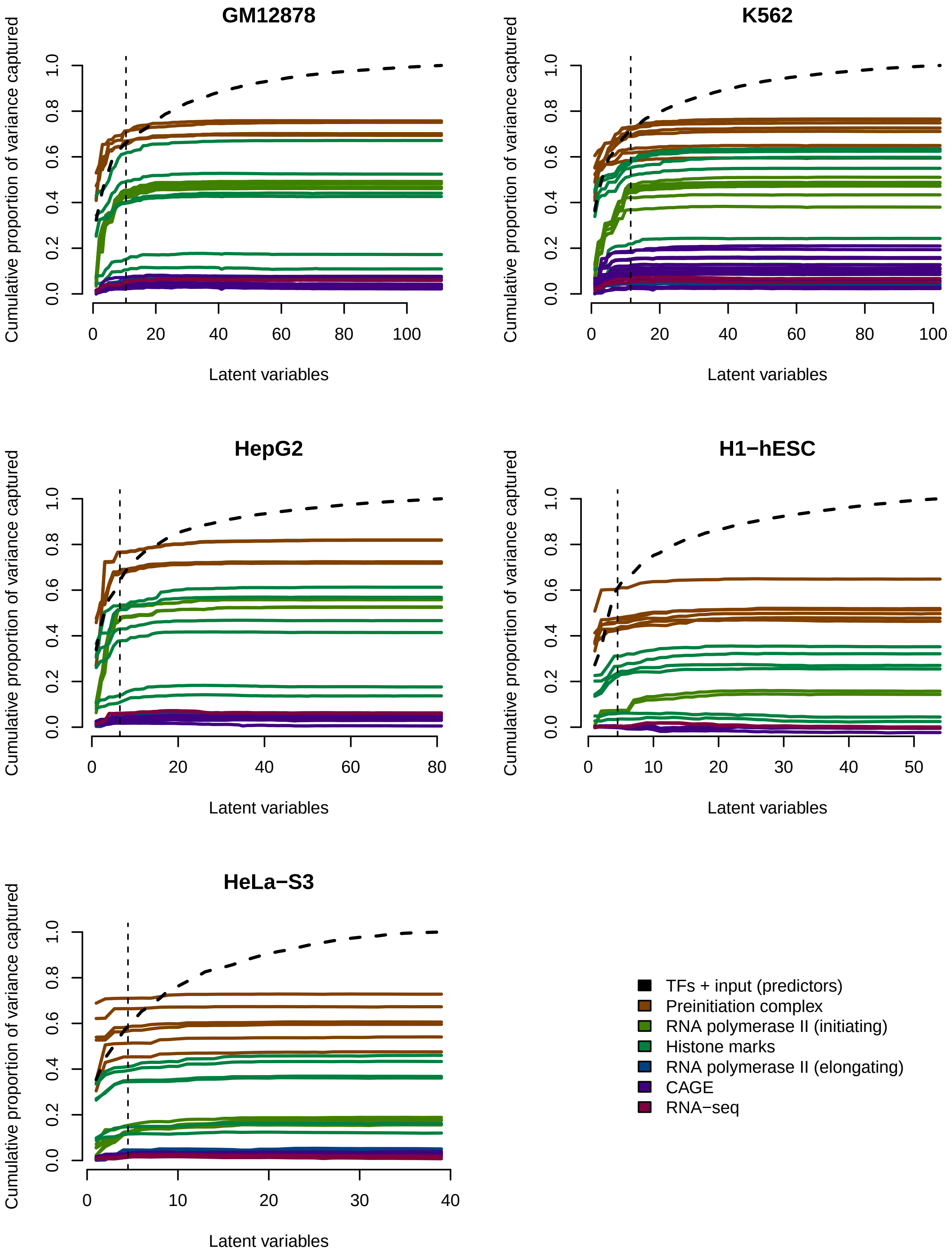

Figure S9: Variance captured by partial least-squares regression analyses.

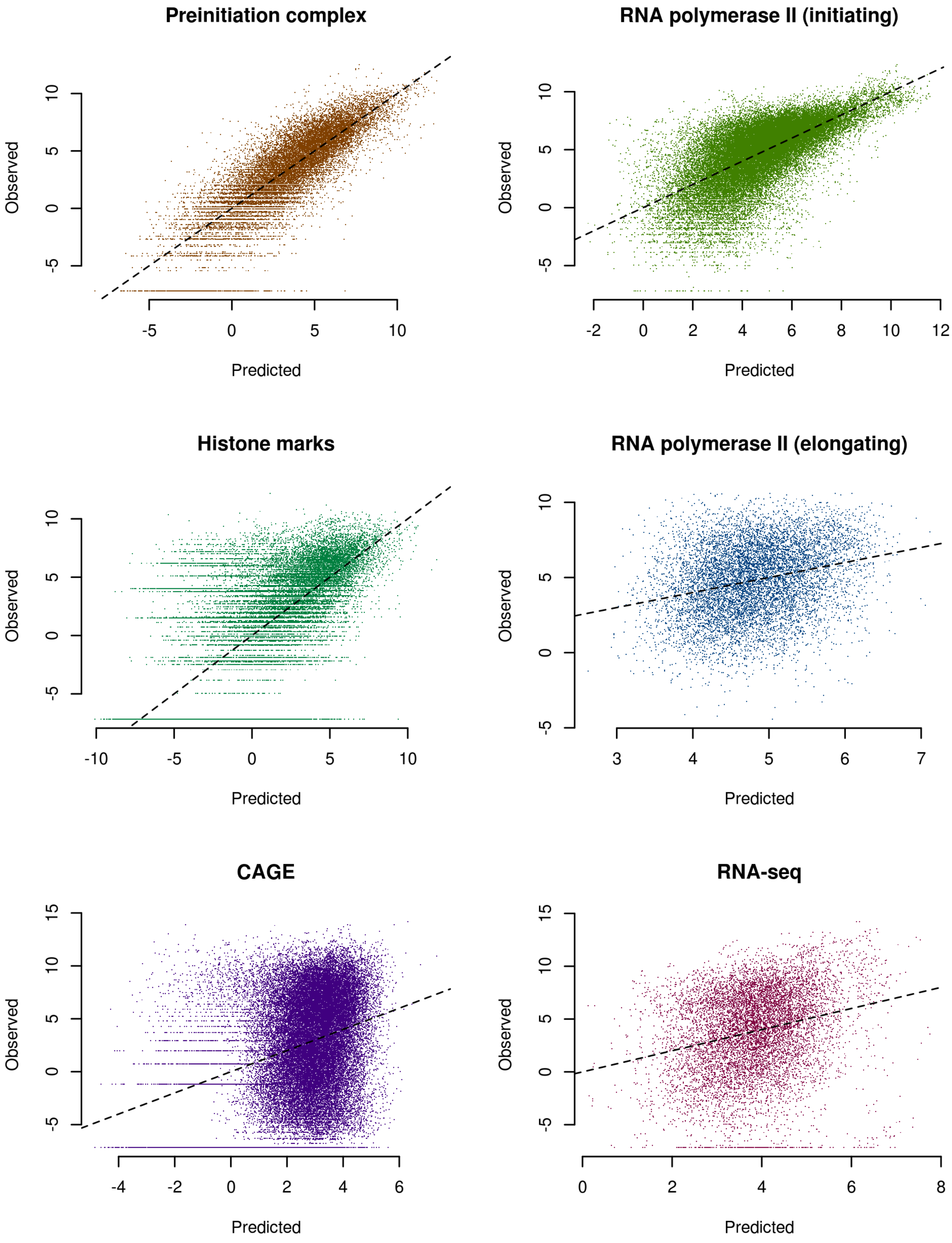
Preinitiation complex
Observed
10
5
0
-5
-5
0
5
10
Predicted
RNA polymerase II (initiating)
Observed
10
5
0
-5
-2
0
2
4
6
8
10
12
Predicted
Histone marks
Observed
10
5
0
-5
-10
-5
0
5
10
Predicted
RNA polymerase II (elongating)
Observed
10
5
0
-5
3
4
5
6
7
Predicted
CAGE
Observed
15
10
5
0
-5
-4
-2
0
2
4
6
Predicted
RNA-seq
Observed
15
10
5
0
-5
0
2
4
6
8
Predicted

Figure S10

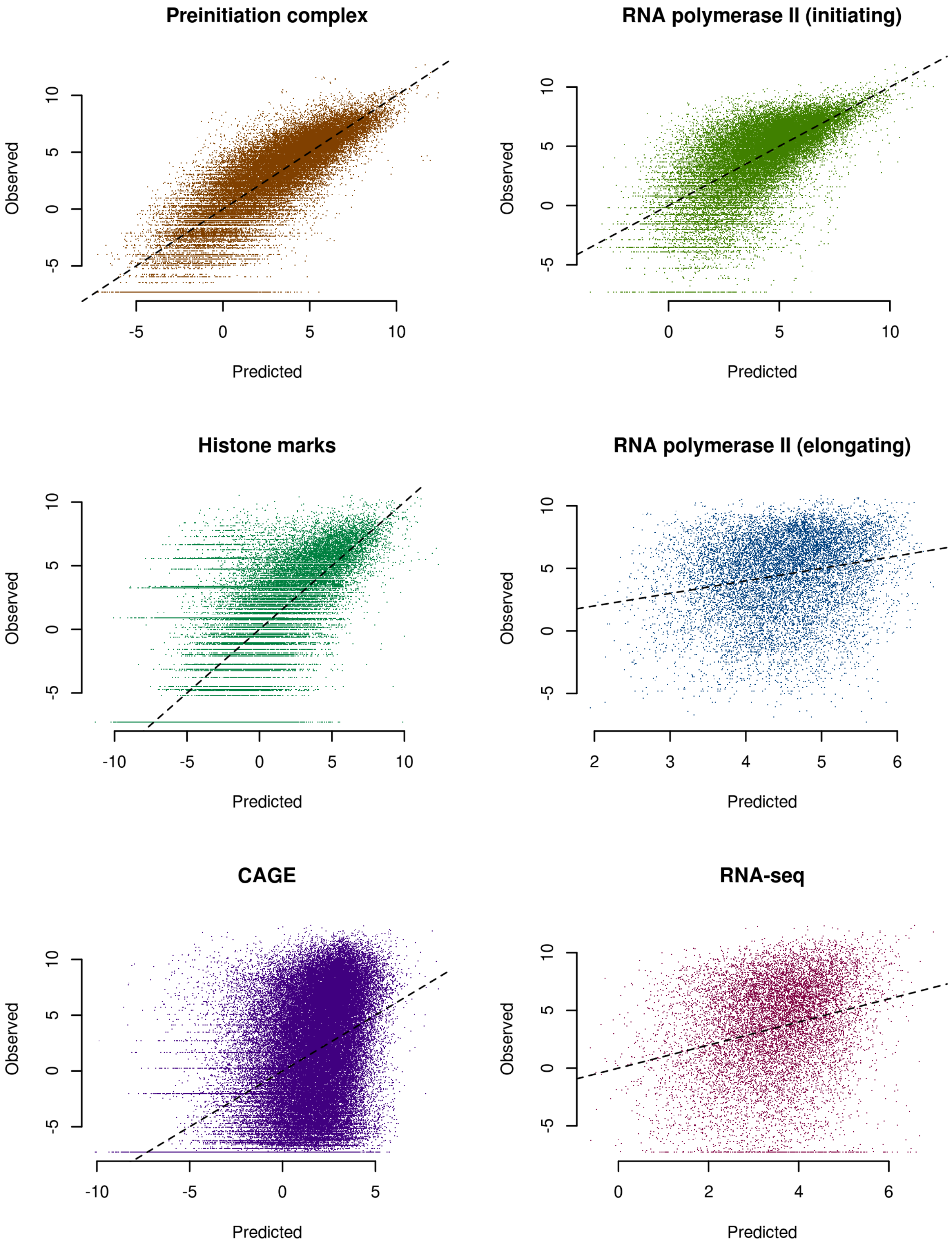
Preinitiation complex
RNA polymerase II (initiating)
Histone marks
RNA polymerase II (elongating)
CAGE
RNA-seq
Observed
Predicted

Figure S10

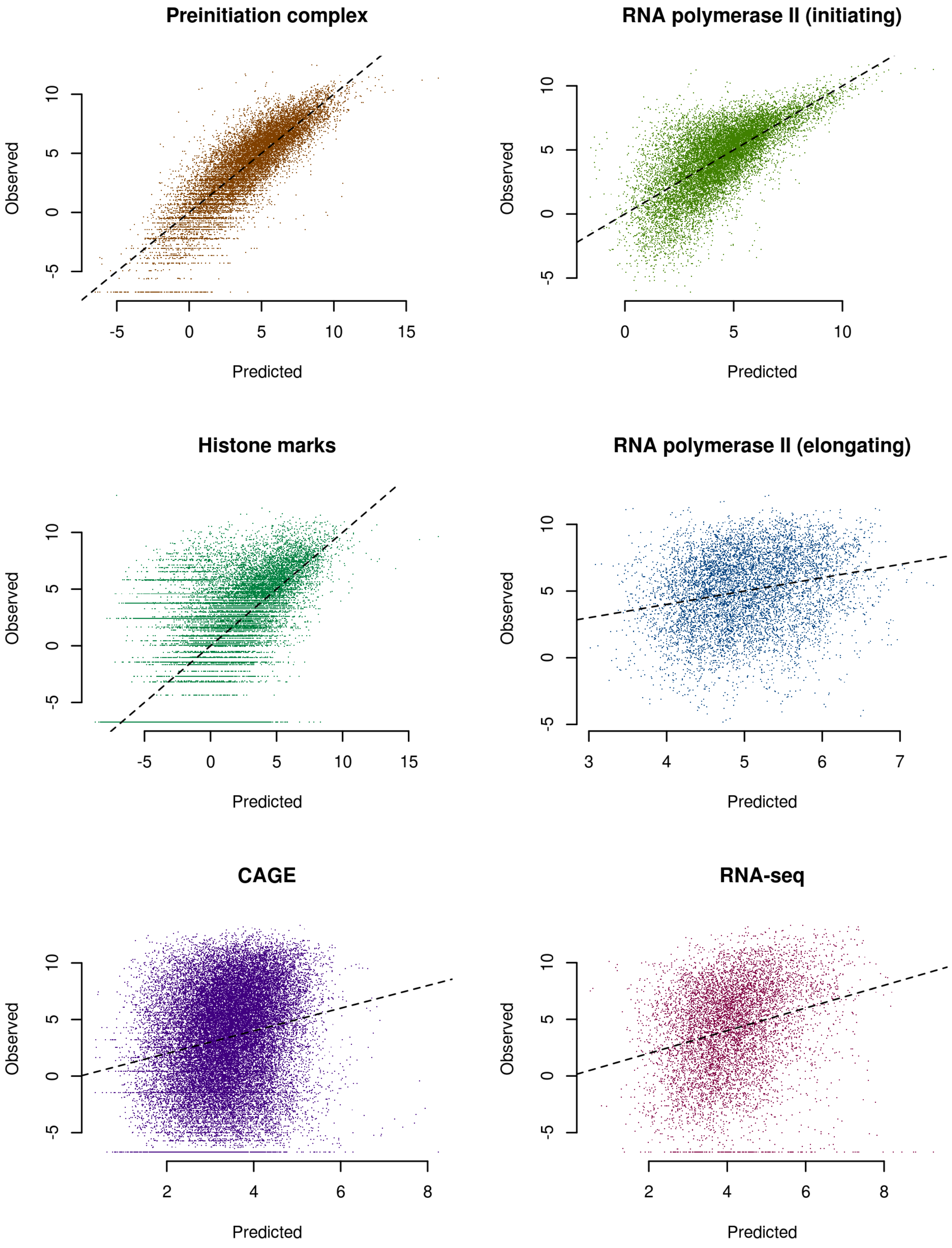

Preinitiation complex
RNA polymerase II (initiating)
Histone marks
RNA polymerase II (elongating)
CAGE
RNA-seq
Observed
Predicted

Figure S10

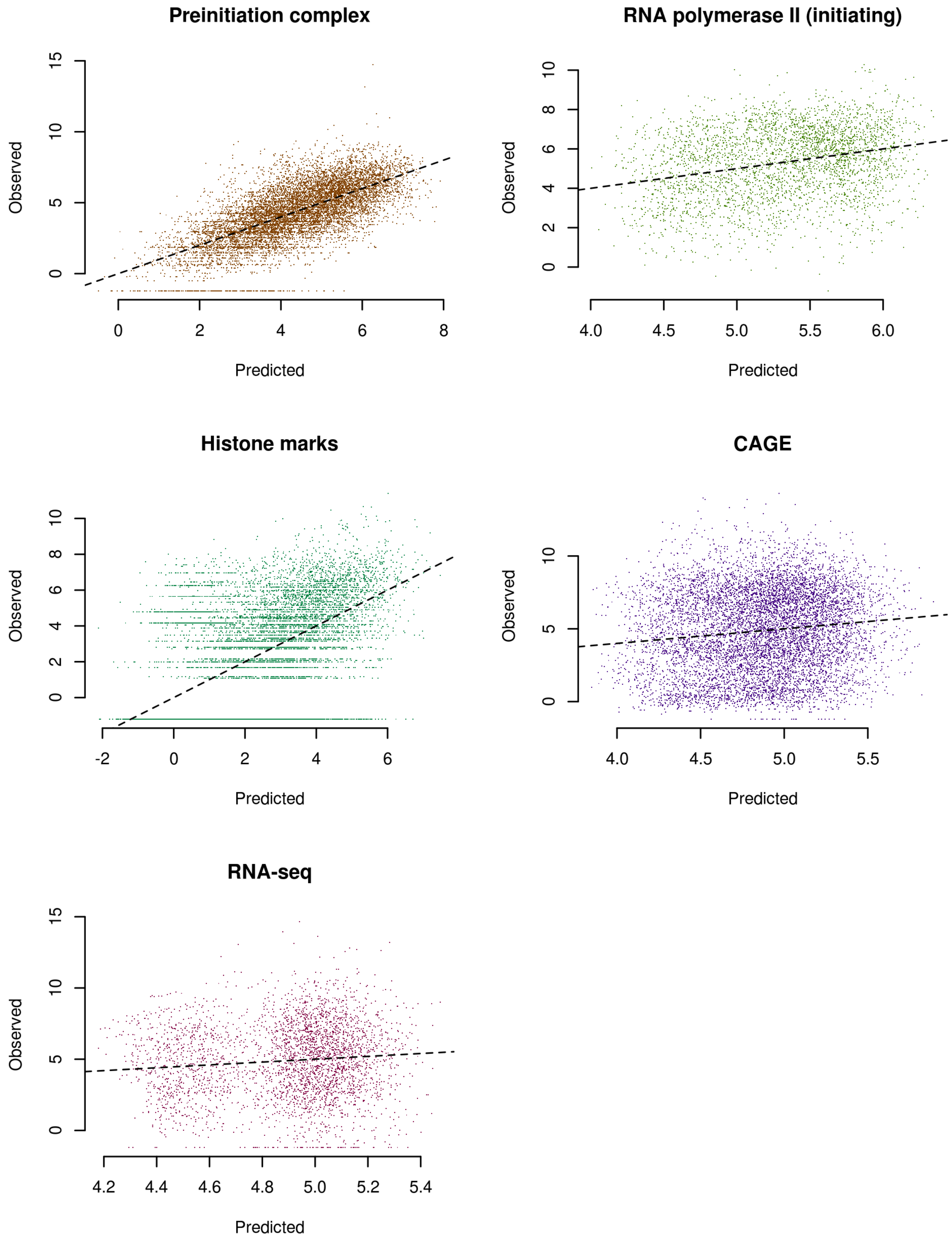
Preinitiation complex
Observed
0
5
10
15
0
2
4
6
8
Predicted
RNA polymerase II (initiating)
Observed
0
2
4
6
8
10
4.0
4.5
5.0
5.5
6.0
Predicted
Histone marks
Observed
0
2
4
6
8
10
-2
0
2
4
6
Predicted
CAGE
Observed
0
5
10
4.0
4.5
5.0
5.5
Predicted
RNA-seq
Observed
0
5
10
15
4.2
4.4
4.6
4.8
5.0
5.2
5.4
Predicted

Figure S10

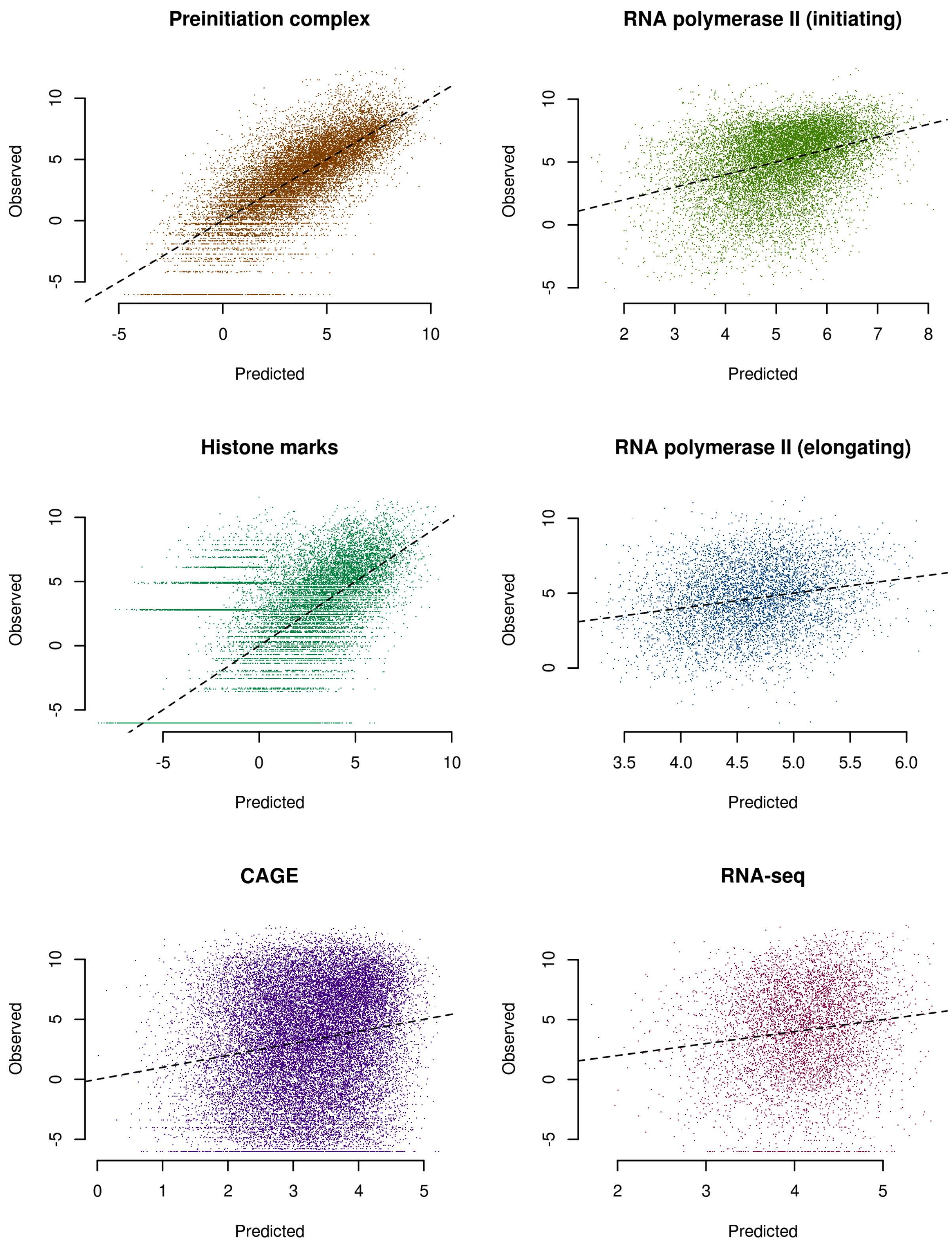

Figure S10: Leave-one-out cross-validation of PLS regression models. S10a: GM12878. S10b: K562. S10c: HepG2. S10d: H1-hESC. S10e: HeLa-S3.
