# Supplementary material for: Transcription-factor occupancy at HOT regions quantitatively predicts RNA polymerase recruitment in five human cell lines
Source: BMC Genomics. 2013 Oct 20;14:720. doi: 10.1186/1471-2164-14-720 (PMC3826616; doi:10.1186/1471-2164-14-720)
Supplement: Additional file 2 — Describes a study validating the UniPeakmethod. [file 1471-2164-14-720-S2.pdf]

Validation of `UniPeak`

Ranking accuracy

We compared the performance of `UniPeak` with that of other ChIP-seq peak callers according to the methods in Wilbanks and Facciotti, 2010 [1]. First, we tested the ranking accuracy on published data sets for the transcription factors FoxA1 [2] and GABP [3]. Sequence reads were aligned to the `hg18` reference assembly by `Bowtie 0.12.9`, and enriched regions were called by `UniPeak 1.0` with default parameters. Enriched regions were ranked by either sequence read count from ChIP or by the logarithm of the p-value from `DESeq 1.10.1`'s two-sided negative-binomial test [4] for ChIP vs. input, signed by direction of the difference. As in Wilbanks and Facciotti, in increments of every 50 regions we calculated the rate of occurrence of each TF's canonical DNA sequence motif (TRANSFAC [5] PSSMs #M01261 and #M00341, respectively) within 250 bp of the density-profile maximum (peak) of each region according to `MAST 4.6.0` [6]. Since rankings are ambiguous for regions with the same read count, we assigned rankings randomly to each group of regions with the same count, and calculated the mean percentages across 1000 replicates of the random ranking.

For FoxA1, `UniPeak`'s performance was competitive with that of other peak callers when using input as a negative control (Figure S1A), as do all peak callers tested in the previous study, and worse without using input. However, the results were the opposite for GABP: `UniPeak` outperformed most other peak callers when disregarding input altogether, but performed much worse when using it as a negative control (Figure S1B). We speculate that this may be because input, rather than a true negative control, actually measures the "background" of chromatin accessibility; and GABP interacts with many other TFs at active promoters [7] while FoxA1 is a "pioneer factor" capable of entering and opening inactive chromatin [8], and binds nucleosomes more stably than accessible DNA [9]. Thus penalizing ChIP enrichment signals for high input enrichment may be counterproductive for some TFs.

Figure S1: Accuracy with respect to motif occurrences. A, B: proportion of peaks within 250 of a canonical motif occurrence for the FoxA1 and GABP data sets, respectively, by rank (50-rank increments). Ranking by ChIP read count or by p-value from a test against input. `UniPeak` results are overlaid on those of other peak callers, from Wilbanks and Facciotti, 2010, figures S2 and S1, respectively [1]. C: Analogous analysis of REST, with results further distinguished between a test against input that combines replicates (discarding variability information) and one that keeps them separate. These results are not directly comparable with Wilbanks and Facciotti figure 6 due to the use of different ChIP-seq data from a different cell line, and different motif PSSMs. D: Distribution of read counts within regions classified by presence (light) or absence (dark) of motifs. Bars show sample means. E: Distribution of signed $\log_{10} p$-values for tests of ChIP signal vs. input signal using unreplicated data or combined replicates (red) or separated replicates (blue), classified by presence (light) or absence (dark) of motifs.

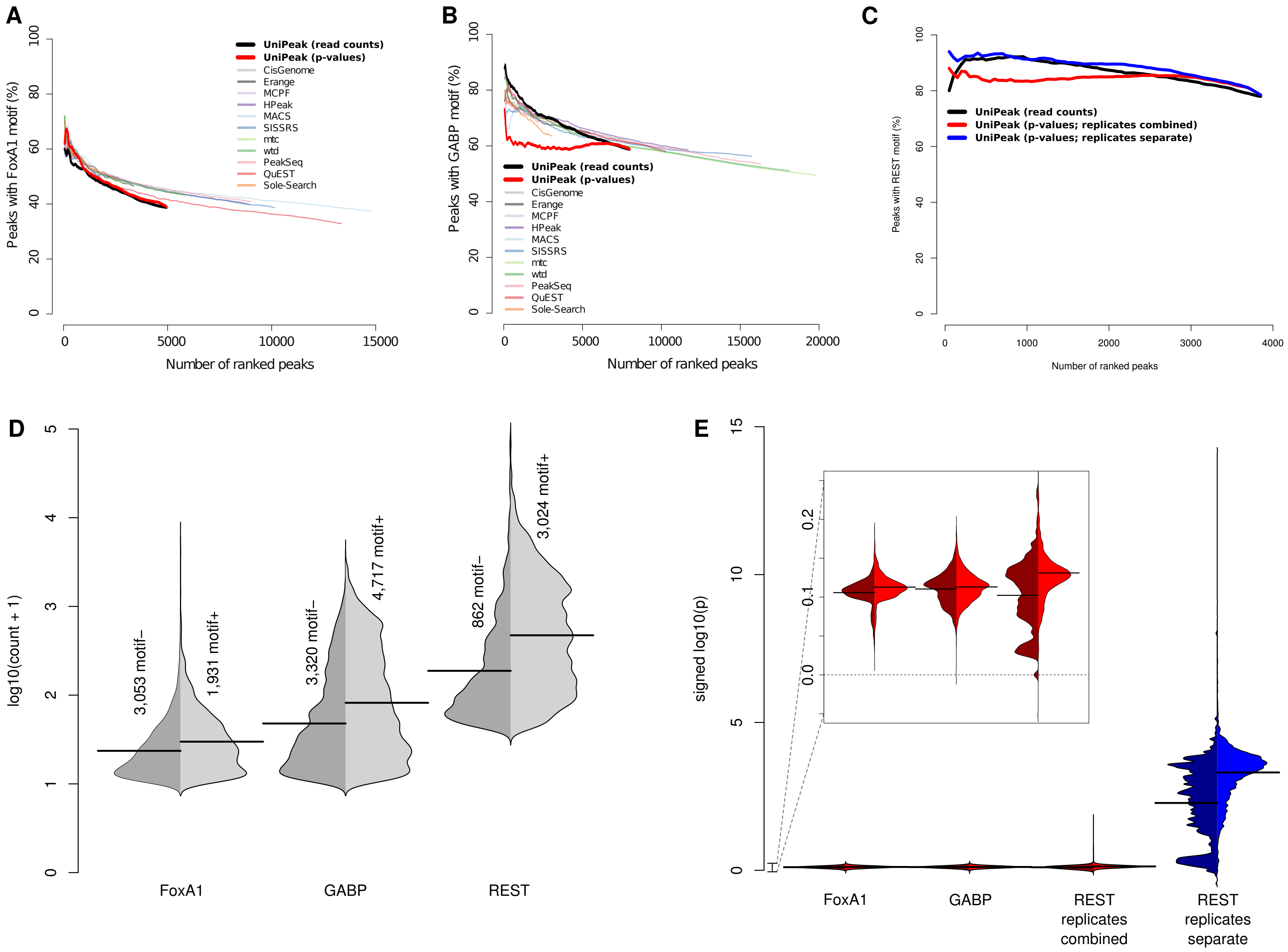
A
UniPeak (read counts)
UniPeak (p-values)
CisGenome
Erange
MCPF
HPeak
MACS
SISSRS
mtc
wtd
PeakSeq
QuEST
Sole-Search
Peaks with FoxA1 motif (%)
Number of ranked peaks
B
Peaks with GABP motif (%)
Number of ranked peaks
C
UniPeak (read counts)
UniPeak (p-values; replicates combined)
UniPeak (p-values; replicates separate)
Peaks with REST motif (%)
Number of ranked peaks
D
log10(count + 1)
3,053 motif−
1,931 motif+
3,320 motif−
4,717 motif+
862 motif−
3,024 motif+
FoxA1
GABP
REST
E
signed log10(p)
FoxA1
GABP
REST replicates combined
REST replicates separate

Figure S1

Use of biological replicates

Although UniPeak performs competitively with other peak callers on the simple data sets from the previous study, its main innovation is that it allows the comparison of any number of samples at a common set of enriched regions. Indeed, even in a simple two-class experimental design it is essential to perform replicates within each class in order to compare meaningful differences between classes with biological variation within classes, but other analysis tools do not effectively take advantage of replicates in their calibration vs. input or provide a straightforward way for the user to substitute a more powerful analysis. Since the data used in the previous analysis were from unreplicated samples, we obtained a much larger data set from ENCODE [10], which comprised four biological replicates of REST ChIP in GM12878 cells and 11 biological replicates of the input control. We performed the same analysis of ranking accuracy as on FoxA1 and GABP, using PSSMs for the "known" full-length REST motif computed by ENCODE [11], except we ranked enriched regions either by their total read count across all ChIP samples, by their signed $\log p$-values from a test for ChIP vs. input where all samples in each class were summed into one (removing the benefit of replication while holding sequencing depth constant), or by their signed $\log p$-values from a test where replicates were treated separately (Figure S1C). Adding replicate information markedly changed the rankings and improved the accuracy of the top ranks.

Quantitative analysis

Another innovation made possible by UniPeak is the treatment of ChIP-seq signals as precisely quantitative data. Simply ranking peaks by their signal destroys the information of their relative strengths, especially since these values come from discrete counts and therefore the rankings are ambiguous. To use the data more effectively, we performed the motif enrichment analysis in a different way: we classified regions by the presence or absence of motifs within 250 bp and compared the enrichment scores between the two classes, where scores were either log-transformed read counts or signed $\log p$-values from the tests described above (Figure S1D). Regions with motifs had higher read counts than regions without motifs (differences in mean $\log_{10}(\text{read count} + 1)$ values: FoxA1 0.10, GABP 0.23, REST 0.40; all significant by one-sided t-test with $p < 2.2 \times 10^{-16}$), and more significant p-values for enrichment relative to input (Figure S1E), with a dramatic improvement from using replicates (differences in mean signed $\log_{10} p$: FoxA1 0.0070, GABP 0.0027, REST replicates combined 0.0288, REST replicates separate 1.0318; all significant by one-sided t-test with $p < 2.2 \times 10^{-16}$ except GABP, with $p = 2.938 \times 10^{-9}$). Furthermore, the distributions of these scores were

multimodal, information that is lost when reducing them to ranks.

Positional precision

Using ChIP-seq peaks within 250 bp of consensus motifs, Wilbanks and Facciotti also compared peak callers for the proximity of detected enrichment peaks to motif centers. We subjected `UniPeak` to the same test under the same conditions for the FoxA1 (Figure S2A) and GABP (Figure S2B) data sets. Again, `UniPeak`'s performance was competitive with other peak callers, though it also detected a bimodal distribution of FoxA1 signals near but not directly on top of motif occurrences. We also compared this positional accuracy by peak with the peak enrichment signals (Figures S2C, D) and found that stronger peaks were more likely to be closer to the motif.

References

1. Wilbanks EG, Facciotti MT: **Evaluation of algorithm performance in ChIP-seq peak detection**. *PLoS ONE* 2010, **5**(7):e11471+, [[http://dx.doi.org/10.1371/journal.pone.0011471]].

2. Zhang Y, Liu T, Meyer C, Eeckhoute J, Johnson D, Bernstein B, Nusbaum C, Myers R, Brown M, Li W, Liu XS: **Model-based Analysis of ChIP-Seq (MACS)**. *Genome Biol* 2008, **9**(9):R137+, [[http://dx.doi.org/10.1186/gb-2008-9-9-r137]].

3. Valouev A, Johnson DS, Sundquist A, Medina C, Anton E, Batzoglou S, Myers RM, Sidow A: **Genome-wide analysis of transcription factor binding sites based on ChIP-seq data.** *Nat Methods* 2008, **5**(9):829–834, [[http://dx.doi.org/10.1038/nmeth.1246]].

4. Anders S, Huber W: **Differential expression analysis for sequence count data**. *Genome Biol* 2010, **11**(10):R106+, [[http://dx.doi.org/10.1186/gb-2010-11-10-r106]].

5. Matys V, Kel-Margoulis OV, Fricke E, Liebich I, Land S, Barre-Dirrie A, Reuter I, Chekmenev D, Krull M, Hornischer K, Voss N, Stegmaier P, Lewicki-Potapov B, Saxel H, Kel AE, Wingender E: **TRANSFAC and its module TRANSCompel: transcriptional gene regulation in eukaryotes.** *Nucleic Acids Res* 2006, **34**(Database issue):D108–D110, [[http://dx.doi.org/10.1093/nar/gkj143]].

6. Bailey TL, Gribskov M: **Combining evidence using p-values: application to sequence homology searches.** *Bioinformatics* 1998, **14**:48–54, [[http://dx.doi.org/10.1093/bioinformatics/14.1.48]].

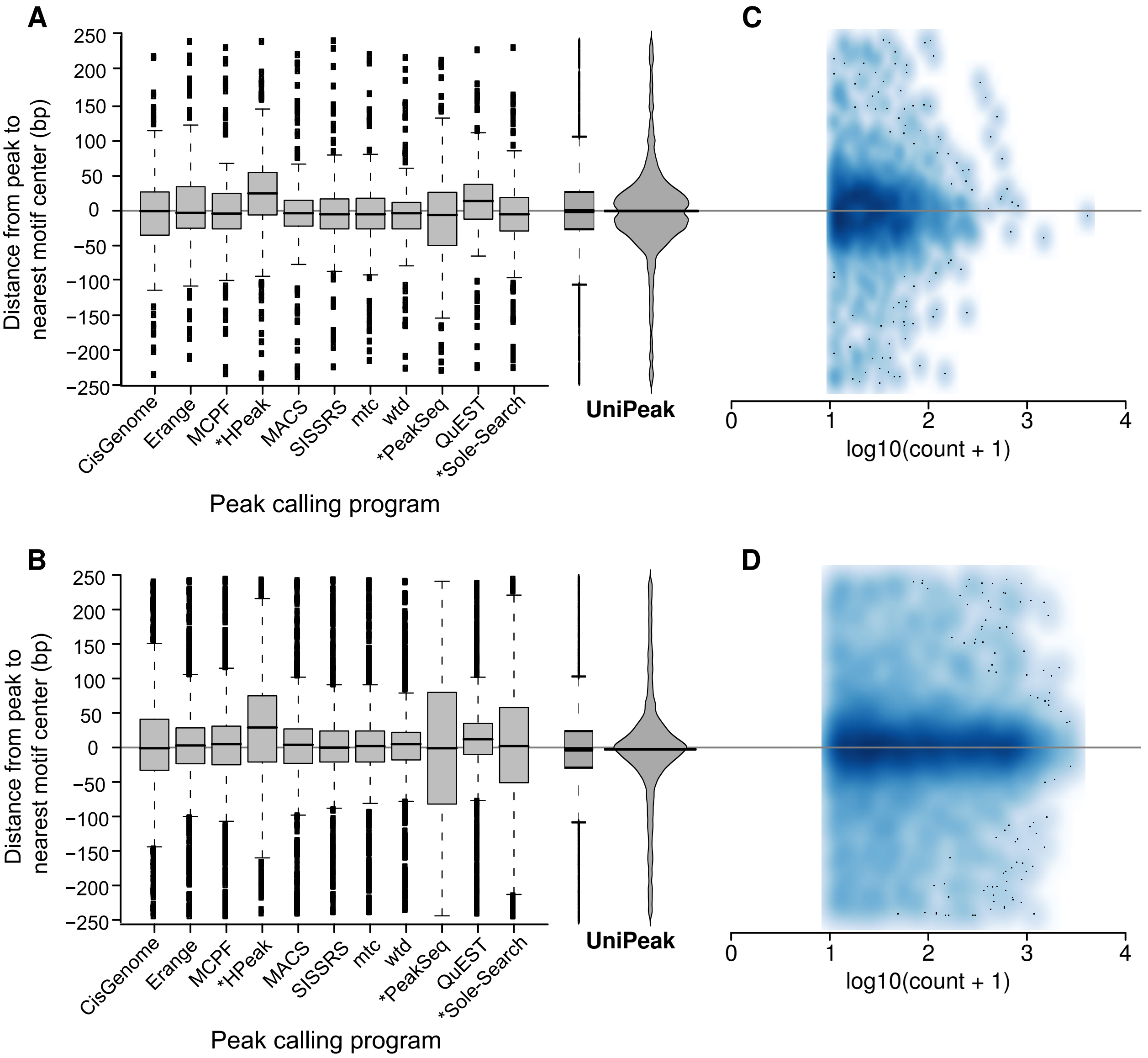

Figure S2: Precision with respect to motif positions. A, B: distribution of enriched-region density maximum (peak) positions relative to centers of motif sites for FoxA1 and GABP, respectively. `UniPeak` results are shown parallel to those of other peak callers, from Wilbanks and Facciotti, 2010, figures 7B and 7C, respectively [1]. C,D: smoothened scatterplots of peak–motif distances vs. peak signal strengths, measured in $\log_{10}$(read count + 1).

7. Rosmarin AG, Resendes KK, Yang Z, McMillan JN, Fleming SL: **GA-binding protein transcription factor: a review of GABP as an integrator of intracellular signaling and protein-protein interactions.** *Blood Cell Mol Dis* 2004, **32**:143–154, [[http://view.ncbi.nlm.nih.gov/pubmed/14757430]].

8. Cirillo LAA, Lin FRR, Cuesta I, Friedman D, Jarnik M, Zaret KS: **Opening of compacted chromatin by early developmental transcription factors HNF3 (FoxA) and GATA-4.** *Mol Cell* 2002, **9**(2):279–289, [[http://view.ncbi.nlm.nih.gov/pubmed/11864602]].

9. Cirillo LA, Zaret KS: **An early developmental transcription factor complex that is more stable on nucleosome core particles than on free DNA.** *Mol Cell* 1999, **4**(6):961–969, [[http://view.ncbi.nlm.nih.gov/pubmed/10635321]].

10. Gerstein MB, Kundaje A, Hariharan M, Landt SG, Yan KK, Cheng C, Mu XJ, Khurana E, Rozowsky J, Alexander R, Min R, Alves P, Abyzov A, Addleman N, Bhardwaj N, Boyle AP, Cayting P, Charos A, Chen DZ, Cheng Y, Clarke D, Eastman C, Euskirchen G, Frietze S, Fu Y, Gertz J, Grubert F, Harmanci A, Jain P, Kasowski M, Lacroute P, Leng J, Lian J, Monahan H, O/'Geen H, Ouyang Z, Partridge EC, Patacsil D, Pauli F, Raha D, Ramirez L, Reddy TE, Reed B, Shi M, Slifer T, Wang J, Wu L, Yang X, Yip KY, Zilberman-Schapira G, Batzoglou S, Sidow A, Farnham PJ, Myers RM, Weissman SM, Snyder M: **Architecture of the human regulatory network derived from ENCODE data**. *Nature* 2012, **489**(7414):91–100, [[http://dx.doi.org/10.1038/nature11245]].

11. Kheradpour P, Kellis M: **ENCODE-motifs: systematic analysis of regulatory motifs associated with transcription factor binding in the human genome**. *Submitted.*
